# A new carcharodontosaurian theropod (Dinosauria: Saurischia) from the Lower Cretaceous of Thailand

**DOI:** 10.1371/journal.pone.0222489

**Published:** 2019-10-09

**Authors:** Duangsuda Chokchaloemwong, Soki Hattori, Elena Cuesta, Pratueng Jintasakul, Masateru Shibata, Yoichi Azuma

**Affiliations:** 1 Northeastern Research Institute of Petrified Wood and Mineral Resources, Nakhon Ratchasima Rajabhat University, Suranaree Subdistict, Mueang, Nakhon Ratchasima District, Nakhon Ratchasima, Thailand; 2 Institute of Dinosaur Research, Fukui Prefectural University, Kenjojima, Matsuoka, Eiheiji–Cho, Fukui, Japan; 3 Fukui Prefectural Dinosaur Museum, Muroko, Terao, Katsuyama, Fukui, Japan; Chinese Academy of Sciences, CHINA

## Abstract

The isolated fossil remains of an allosauroid theropod from the Lower Cretaceous Khok Kruat Formation of Khorat, Thailand, are described in this study. Detailed observations support the establishment of a new allosauroid, *Siamraptor suwati* gen. et sp. nov. This new taxon is based on a composite cranial and postcranial skeleton comprising premaxilla, maxilla, jugal, surangular, prearticular, articular, vertebrae, manual ungual, ischium, tibia, and pedal phalanx. It is distinguished from other allosauroids by characters such as a jugal with straight ventral margin and dorsoventrally deep anterior process below the orbit, a surangular with a deep oval concavity at the posterior end of the lateral shelf and four posterior surangular foramina, a long and narrow groove along the suture between the surangular and the prearticular, an articular with a foramen at the notch of the suture with the prearticular, an anterior cervical vertebra with a pneumatic foramen (so-called ‘pleurocoel’) excavating parapophysis, and cervical and posterior dorsal vertebrae penetrated by a pair of small foramina bilaterally at the base of the neural spine. The presence of a huge number of camerae and pneumatopores in cranial and axial elements reveals a remarkable skeletal pneumatic system in this new taxon. Moreover, the phylogenetic analyses revealed that *Siamraptor* is a basal taxon of Carcharodontosauria, involving a new sight of the paleobiogeographical context of this group. *Siamraptor* is the best preserved carcharodontosaurian theropod in Southeast Asia, and it sheds new light on the early evolutionary history of Carcharodontosauria.

## Introduction

The basal tetanuran clades Allosauroidea and Megalosauroidea appeared by the Middle Jurassic and were soon represented by large-bodied taxa. These two clades are key to understanding Middle Jurassic-early Late Cretaceous dinosaurian ecosystems, in which they comprised almost all large predators over a span of approximately 85 million years. Although large theropod records from Early Cretaceous are scarce in Asia [1], some important specimens have been recovered in recent years [2–10]. Allosauroidea, a clade of large-bodied theropod dinosaurs that ranged from the Middle Jurassic until the Late Cretaceous, has been the subject of extensive phylogenetic study (e.g. [11–14]). Among them, Carcharodontosauria is the most inclusive clade, comprising *Carcharodontosaurus saharicus* and *Neovenator salerii*, but not *Allosaurus fragilis* or *Sinraptor dongi* [14], which was established as a replacement of the original definition of the Carcharodontosauridae named by Stromer [15] defined as *C*. *saharicus*, and all the taxa share a more recent common ancestor with it than with *A*. *fragilis* or *S*. *dongi* [1]. Coincidently, Carcharodontosauridae was redefined as the most inclusive clade comprising *C*. *saharicus* but not *N*. *salerii*, *A*. *fragilis* or *S*. *dongi* [1]. However, Asian carcharodontosaurs have been exceptionally poorly known so far in the Early to mid-Cretaceous of Asia [1,9].

**Fig 1:**
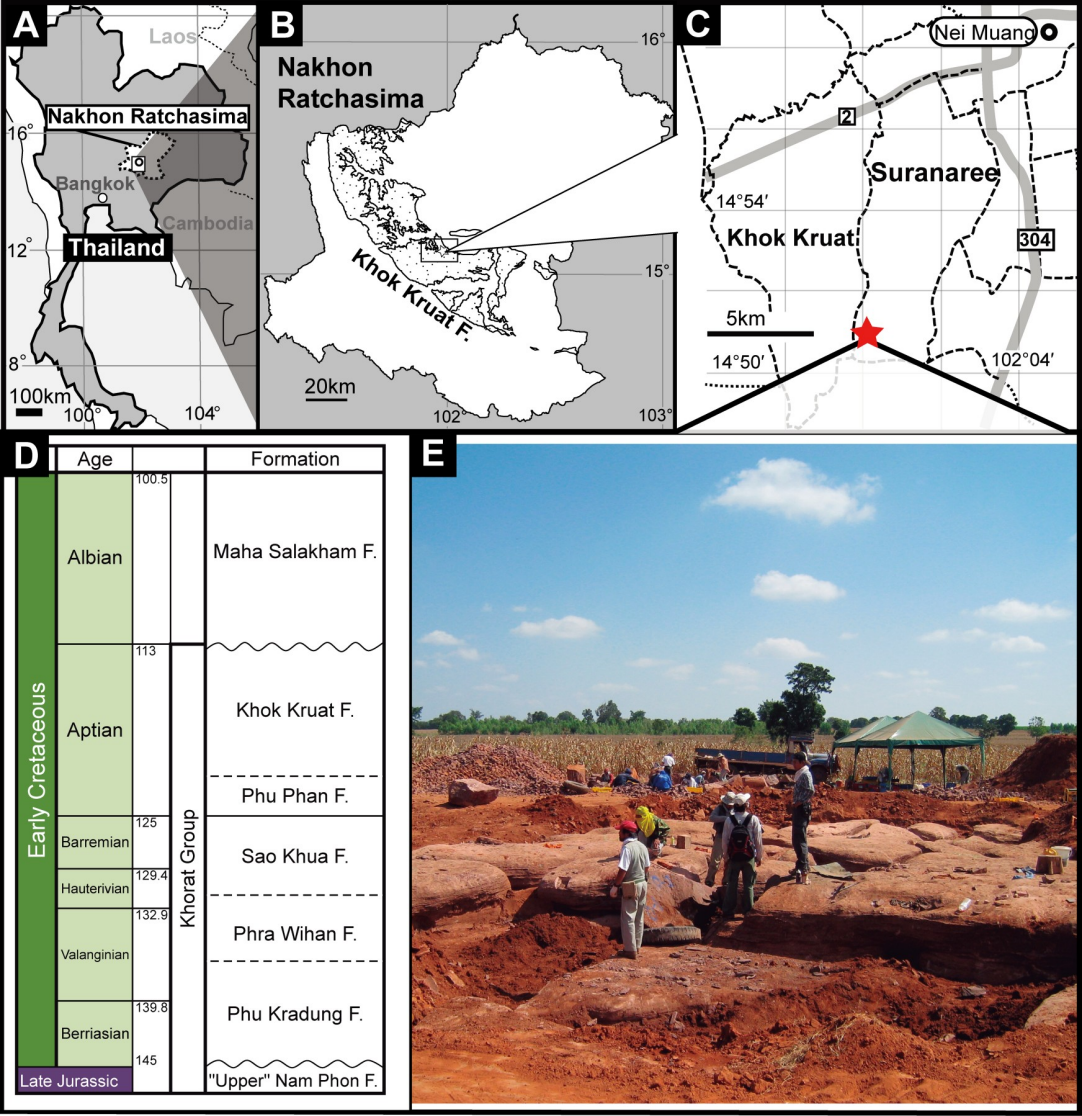
Locality map of new theropod material and stratigraphy of Khorat Group. A, map of Nakhon Ratchasima Province, Thailand; B, distribution map of the Khok Kruat Formation in Nakhon Ratchasima Province (modified from the Geological map of Thailand, Department of Mineral Resources); C, enlarged locality map of Suranaree and Khok Kruat subdistricts with the subdistrict boundaries; D, a photograph of the excavation site; E, stratigraphic column of the Khorat Group [28]. A red-colored star indicates the new theropod locality, the dotted lines indicate the subdistrict boundaries, and the grey-colored lines indicate the roads in C, respectively.

In the last decade, Thailand has yielded a huge number of Mesozoic non-marine fossil vertebrates, ranging from the Late Triassic to the late Early Cretaceous [16]. Nevertheless, dinosaur remains from the Lower Cretaceous Khok Kruat Formation have hitherto been scarce, and only iguanodontian ornithopods have been described based on isolated remains [17–19]. In this study, a new theropod taxon is described based on extensive cranial and postcranial materials collected in a locality of the Khok Kruat Formation of the Japan-Thailand Dinosaur Project (abbreviated as JTDP [19]; Fig 1).

**Fig 2:**
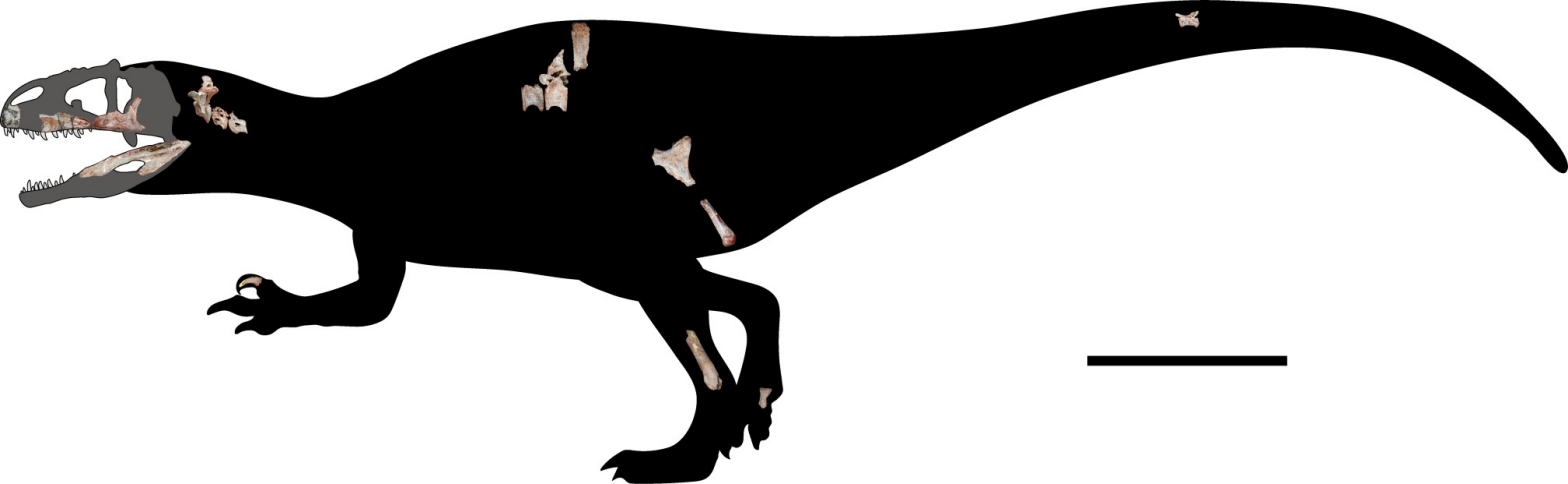
Skeletal reconstruction of *Siamraptor suwati*. Cranial elements were scaled to fit in with the holotype (surangular). Scale bar equals 1 m.

In this study, this new taxon is considered as a member of Allosauroidea in this study based on several features of extensive skull elements, axial material, ischium, tibia, manual ungual, and a pedal phalanx (Fig 2). All the measurements of bones are shown in Table 1. The osteological description of this taxon shows a notorious skeletal pneumaticity in the skull and axial elements, which presents some features that are similar to those observed in derived allosauroids such as *Aerosteon* [21] and *Murusraptor* [22], and some particular features, like the presence of pneumatic foramina in the surangular, camerate structures in the cervical vertebrae, or foramina in the base of cervical and dorsal neural spines. The results of the phylogenetic analysis indicate that the new taxon is a new basal member of Carcharodontosauria from the Early Cretaceous of Southeast Asia. Although other carcharodontosaurians have also been reported from Asia such as *Fukuiraptor* [23], *Shaochilong* [5], *Kelmayisaurus* [24] and an indeterminate and fragmentary carcharodontosaurid from Thailand [25], this is the first report of the presence of a more basal carcharodontosaurian theropod in this area. In combination with the presence of carcharodontosaurian materials in the Upper Jurassic of Portugal [26] and Tanzania [12,27], this study also indicates the wide distribution of Carcharodontosauria during the Upper Jurassic and Early Cretaceous, which is consistent with previous paleobiogeographic studies about Mesozoic faunal interchanges [20,28].

**Table 1:** Measurements of the described materials of *Siamraptor suwati*.

Element	Specimen Number	Side	Length (mm)	Width (mm)	Height (mm)
Premaxilla	NRRU-F01020001	Right	112	40	150
Premaxilla	NRRU-F01020002	Right	110	31	139
Premaxilla	NRRU-F01020003	Right	104	33	121
Maxilla	NRRU-F01020004	Right	147	37	76
Maxilla	NRRU-F01020005	Left	115	32	84
Jugal	NRRU-F01020006	Left	210	20	135
Posterior part of the mandible (surangular, prearticular, articular)	NRRU-F01020007	Left	250	61	125
Posterior part of the mandible (surangular, prearticular, articular)	NRRU-F01020008	Right	555	126	124
Posterior part of the mandible (surangular, prearticular, articular)	NRRU-F01020009	Left	237	92	133
Posterior part of the mandible (surangular, prearticular)	NRRU-F01020010	Left	148	47	62
Anterior cervical vertebra	NRRU-F01020011	-	112	140	193
Middle cervical vertebra	NRRU-F01020012	-	89	91	114
Middle cervical vertebra	NRRU-F01020013	-	78	99	107
Posterior dorsal vertebra	NRRU-F01020014	-	130	116	281
Posterior dorsal centrum	NRRU-F01020015	-	89	85	127
Posterior dorsal neural spine	NRRU-F01020016	-	246	39	92
Middle caudal vertebra	NRRU-F01020017	-	107	35	79
Manual ungual	NRRU-F01020018	?	115	19	43
Right ischium (distal part)	NRRU-F01020019	Right	265	45	77
Right ischium (proximal part)	NRRU-F01020020	Right	250	41	214
Tibia	NRRU-F01020021	Right	305	61	180
Pedal phalanx IV-1	NRRU-F01020022	Left	93	44	57

### Geological setting

Cretaceous sediments in Thailand are composed mostly of non-marine deposits. The Khorat Group is the most fossiliferous strata in Southeast Asia, and is distributed on the Khorat Plateau in northeastern Thailand (Fig 1A and 1B; e.g. [29,30]). The Khok Kruat Formation is the uppermost unit of the Khorat Group and is widely distributed in the Khorat Basin of northeastern Thailand (Fig 1A, 1B and 1E; [30]). This formation is 430–700m thick and consists mainly of reddish-brown siltstones and sandstones [30,31]. The main locality of the dinosaur fossils of the Khok Kruat Formation, where the Japan-Thailand Dinosaur Project (JTDP) has worked, is located in Ban Saphan Hin, the Suranaree Sub-district, northwest of the Muang District, Nakhon Ratchashima Province (Fig 1C and 1D). Yukawa et al. [32] recognized three sedimentary facies of channel and bar, crevasse-splay and floodplain in the Khok Kruat Formation. The bone-bearing beds consist of medium- to coarse-grained sandstones and conglomerates containing clasts of clay rip-up pebble and rounded calcareous nodule granules with planar, cross and large scaled epsilon-type cross laminations. This lithofacies represents the channel and bar deposits. The crevasse-splay facies are characterized by poorly sorted fine-grained massive sandstone and layers of parallel laminated very fine-grained sandstones, while the floodplain facies are composed of massive mudstones with the paleosol [32].

The sedimentary environment of the Khorat Group represents the braided to meandering river systems [30], and the Khok Kruat Formation consists of meandering river deposits in the Khorat Basin. Yukawa et al. [32] interpreted that the deposits at the dinosaur fossil locality show point bars, channels and floodplain facies. Meesook [30] reconstructed the Cretaceous paleoclimate based on paleosols in deposits of the Khorat Group because the paleobotanical records are sparse. The paleoclimate represents a semi-arid to humid climate in the Khorat Basin, and, subsequently, again a semi-arid climate in the Sao Khua Formation, and slightly humid in the Phu Phan Formation. The Khok Kruat Formation was deposited under the semi-arid climate. However, Amiot et al. [33] suggest that the Khorat Basin was formed under a subtropical or tropical climate and a semi-humid condition.

The Phu Kradung, Sao Khua and Phra Wihan Formations are assigned to the Early Cretaceous (Berriasian–Barremian). However, the lower part of the Phu Kradung Formation may include the Upper Jurassic, based on dinosaur fossils [29,34]. Although the geological age of the Khok Kruat Formation is not definitively decided due to the lack of age-diagnostic fossils, the Aptian age is widely accepted based on the palynological data, the occurrences of the fresh water hybodont shark *Thaiodus ruchae*, and the basal ceratopsian *Psittacosaurus sattayaraki*. The overlying Maha Sarakham Formation is inferred to belong to the Albian–Cenomanian age [10,29,35,36]. In this study, we adopt the Aptian age for the Khok Kruat Formation.

### Nomenclatural acts

The electronic edition of this article conforms to the requirements of the amended International Code of Zoological Nomenclature, and hence the new names contained herein are available under that Code from the electronic edition of this article. This published work and the nomenclatural acts it contains have been registered in ZooBank, the online registration system for the ICZN. The ZooBank LSIDs (Life Science Identifiers) can be resolved and the associated information viewed through any standard web browser by appending the LSID to the prefix “http://zoobank.org/”. The LSID for this publication is: urn:lsid:zoobank.org:pub:8702A94C-F2B5-4A4D-9454-52220F2D1C85. The electronic edition of this work was published in a journal with an ISSN and has been archived and is available from the following digital repositories: PubMed Central, LOCKSS.

## Materials and methods

The specimens described here (NRRU-F01020001–NRRU-F01020022) are housed at public and permanent repository in the collection of the Northeastern Research Institute of Petrified Wood and Mineral Resources, Nakhon Ratchasima Rajabhat University, Thailand, and are accessible to all researchers. No permits were required for the described study, which complies with all the relevant regulations. The excavation and collection of fossil remains were agreed with the landowner and were officially reported to the Department of the Mineral Resources, Thailand.

Institutional abbreviations; BMNH, Natural History Museum, London, England, UK; BYU, Brigham Young University, Provo, UT, USA; DINO, Dinosaur National Monument, Vernal, UT, USA; IVPP, Institute of Paleontology and Paleoanthropology, Beijing, China; FPDM, Fukui Prefectural Dinosaur Museum, 51–11 Muroko, Terao, Katsuyama, Fukui, 911–8601, Japan; MCF, Museo Municipal “Carmen Funes”, Plaza Huincul, Argentina; MIWG, Dinosaur Isle Museum of Isle of Wight, Sandown, UK; MCCM, Museo de Ciencias de Castilla-La Mancha, Cuenca, Spain, now MUPA, Museo de Paleontología de Castilla-La Mancha; MPEF, Museo Paleontológico “Egidio Feruglio”, Trelew, Argentina; MUCPv-CH, Museo de la Universidad del Comahue, Colección Chocón, Villa El Chocón, Argentina; NCSM, North Carolina Museum of Natural Science, Raleigh, NC, USA; OMNH¸ Sam Noble Oklahoma Museum of Natural History, Norman, OK, USA; UMNH, Natural History Museum of Utah/University of Utah, Salt Lake City, UT, USA.

### Systematic paleontology

Dinosauria Owen, 1842 [37]

Theropoda Marsh, 1881 [38]

Tetanurae Gauthier, 1986 [39]

Allosauroidea Marsh, 1878 [40]

Carcharodontosauria Benson, Carrano and Brusatte, 2010 [14]

*Siamraptor* gen. nov.

urn:lsid:zoobank.org:act:70FBCCF0-1547-420F-AC6E-FA0BE799CAE5

*Siamraptor suwati* sp. nov.

urn:lsid:zoobank.org:act:46E1572B-54CE-4276-A6C0-8FF783D75954

#### Etymology

*Siam* (Latin): in reference to Thailand; *raptor* (Latin): meaning a robber; *suwati*: in honour of Mr. Suwat Liptapanlop, who supports and promotes the work of the Northeastern Research Institute of Petrified Wood and Mineral Resources.

#### Diagnosis

Allosauroid theropod with the following autapomorphies among allosauroids: Jugal with straight ventral margin, and dorsoventrally deep anterior process below the orbit; surangular with a deep oval concavity at the posterior end of the lateral shelf and four posterior surangular foramina; long and narrow groove along the suture between surangular and prearticular; articular with a foramen at the notch of the suture with prearticular; anterior cervical vertebra with an additional pneumatic foramen excavating parapophysis; cervical and posterior dorsal vertebra penetrated by a pair of small foramina bilaterally at the base of neural spine.

#### Holotype

An articulated posterior half of the right mandible comprising the surangular, prearticular, and articular (NRRU-F01020008).

#### Referred materials

Disarticulated cranial and postcranial elements from at least three individuals; three right premaxillae (NRRU-F01020001–F01020003), a right (NRRU-F01020004) and a left (NRRU-F01020005) maxillae, a left jugal (NRRU-F01020006), two posterior parts of the left mandible comprising the surangular, prearticular, and articular (NRRU-F01020007, F01020009), a posterior part of the left mandible comprising the surangular and prearticular (NRRU-F01020010), three anterior cervical vertebrae (NRRU-F01020011–F01020013), three posterior dorsal vertebrae (NRRU-F01020014–F01020016), a middle caudal vertebra (NRRU-F01020017), a manual ungual (NRRU-F01020018), a right ischium (NRRU-F01020019 and F01020020), a distal part of the right tibia (NRRU-F01020021), and a left pedal phalanx IV-1 (NRRU-F01020022). All of these are materials comparable to Allosauroidea that were found in a small area (125 m x 160 m) of a single layer of a single locality, and the overlapping materials exhibit the same diagnostic features.

#### Locality and horizon

In Ban (meaning “village”) Saphan Hin, Suranaree Subdistrict, Muaeng Nakhon Ratchasima District, Nakhon Ratchasima Province, Thailand. Lower Cretaceous (Aptian) Khok Kruat Formation.

### Description

#### Premaxilla

**Fig 3:**
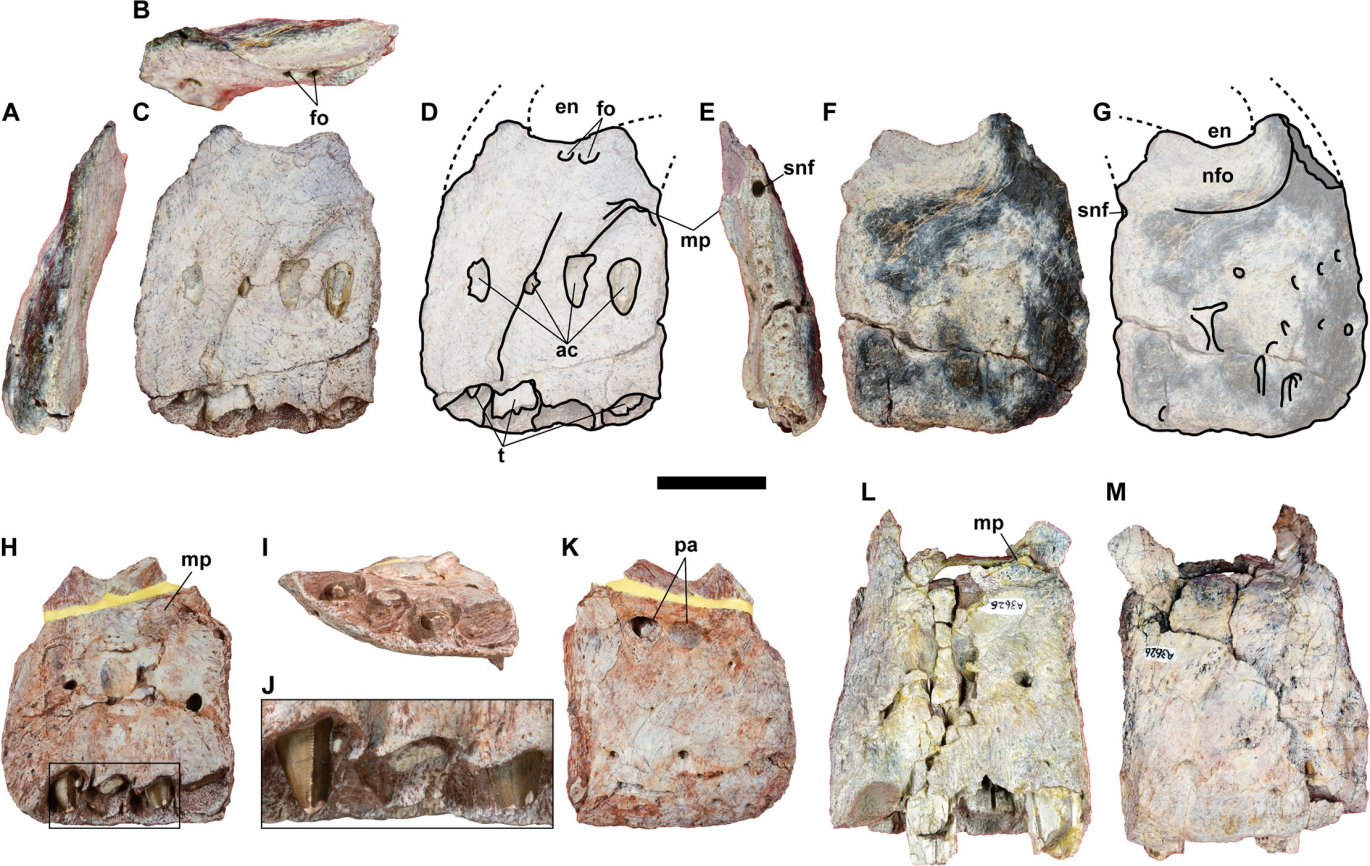
**Right premaxillae, NRRU-F01020003 (A–G), F01020002 (H–K) and F01020001 (L, M) in anterior (A), dorsal (B), medial (C, D, H, L), posterior (E), lateral (F, G, K, M) and alveolar (I) views.** The rectangle in H indicates the area magnified in J to show premaxillary teeth. Abbreviations: ac, alveolar channel; en, external naris; fo, foramen; mp, maxillary process; nfo, nasal fossa; pa, possible pathology; snf, subnarial fossa; t, tooth. Scale bar equals 50 mm.

The premaxillary body is preserved on each of the three right premaxillae (NRRU-F01020001–F01020003; Fig 3). The proportion of the main premaxillary body is approximately as anteroposteriorly long as dorsoventrally deep. The anterior margin of the premaxillary body is almost vertical unlike carcharodontosaurians, which have a posterodorsally inclined margin [11]. The narial fossa is well developed and situated just ventral to the external naris, unlike *Concavenator* and *Acrocanthosaurus* [41,42], in which the fossa is situated anterior to the external naris. Only the basalmost part of the supranarial and subnarial processes are preserved in NRRU-F01020001. As preserved, the supranarial process is dorsally directed and the subnarial one is posterodorsally directed.

The posterior surface for the contact with the maxilla is preserved but somewhat eroded in each specimen. In lateral view, the contact is almost perpendicular to the ventral margin of the bone, and is almost straight, as in *Allosaurus* [43], rather than convex, as in *Sinraptor* [44] and *Acrocanthosaurus* [41]. There is a marked subnarial foramen in the contact surface for the maxilla, which makes a small notch in lateral view. This foramen is deeply excavated and wide in posterior view, although is not an expanded channel as those observed in spinosaurids [12]. Ventral to this subnarial foramen, there are smaller foramina in a row on the posterior surface of the premaxilla. The lateral surface is almost smooth, lacking extensive external sculpturing, unlike derived carcharodontosaurids [11]. However, several foramina and ventrally-directed grooves puncture the lateral surface of the premaxillary body, these foramina and grooves being as abundant and developed as in other allosauroids, such as *Allosaurus* (e.g. UMNH-VP 9248, 9250, 6502) and *Neovenator* (MIWG 6348).

In NRRU-F01020003, just ventral to the external naris, there are two foramina on the medial surface. These foramina are not observed in the other two specimens because of the breakage in the corresponding region. Although only one large foramen is present in *Sinraptor* [44] and *Neovenator* (MIWG 6248), the number of foramina varies among the specimens of *Allosaurus* from one to three, so that two foramina are also seen in some specimens (UMNH-VP 6504, 9238, 9252). The presence of these foramina is common in most large theropods [45]. The maxillary processes are well developed on the medial surface of NRRU-F01020002 and NRRU-F01020003 as a posterodorsally-oriented flange beneath the base of the subnarial process, but its posteromedial end is broken in each specimen and missing in NRRU-F01020001. The location of this process is relatively higher than in *Sinraptor* [44], as in *Allosaurus* (e.g. UMNH-VP 9238, 9248, 6502, 9252) and *Neovenator* (MIWG 6348). Dorsally, the medial surface between the external naris and the maxillary process is excavated to form a broad posteriorly-oriented groove for the contact with the anteromedial process of the maxilla, as in *Allosaurus* [43] and *Neovenator* [45]. More ventrally, the medial surface has four alveolar channels as openings in a row at the mid-height of the maxillary body with visible unerupted teeth.

Four alveoli are present in the premaxilla. NRRU-F01020003 has three incomplete teeth, NRRU-F01020002 has two complete erupted teeth, and NRRU-F01020001 has three incomplete erupted teeth and two unerupted teeth. The shape of each alveolus is oval, and the size is similar to each other. Each of these alveoli has the alveolar channel on the medial surface of the premaxilla.

In premaxillary teeth in all the premaxillae, both the anterior and posterior carinae are in the lingual side as in Averostra [46] but unlike the symmetrical carinae in Carcharodontosauridae [47]. Both carinae have serration and have two to three denticles per millimeter in the middle region. In NRRU-F01020002, the first and third teeth show mesial and distal carinae situated at the mesial and distal margins of the lingual surface to adopt a D-shaped cross section at its mid-crown. In NRRU-F01020003, the mid-crown cross section of the posteriormost teeth is elliptical and transversally compressed. The teeth seem to not present marked enamel wrinkles. The interdental plates are completely ossified. In ventral view, the premaxilla is almost straight, indicating an acute morphology of the symphysis, although the specimen lacks the left premaxilla in articulation.

In NRRU-F01020002, there is an unusual oval opening below the posteroventral margin of the narial fossa in lateral view. Anterior to this fossa, there is an oval, slightly swelling area composed of an apparently different, abnormal bone tissue. These opening and swelling are absent in the other two specimens; therefore, they possibly indicate pathologies.

#### Maxilla

**Fig 4:**
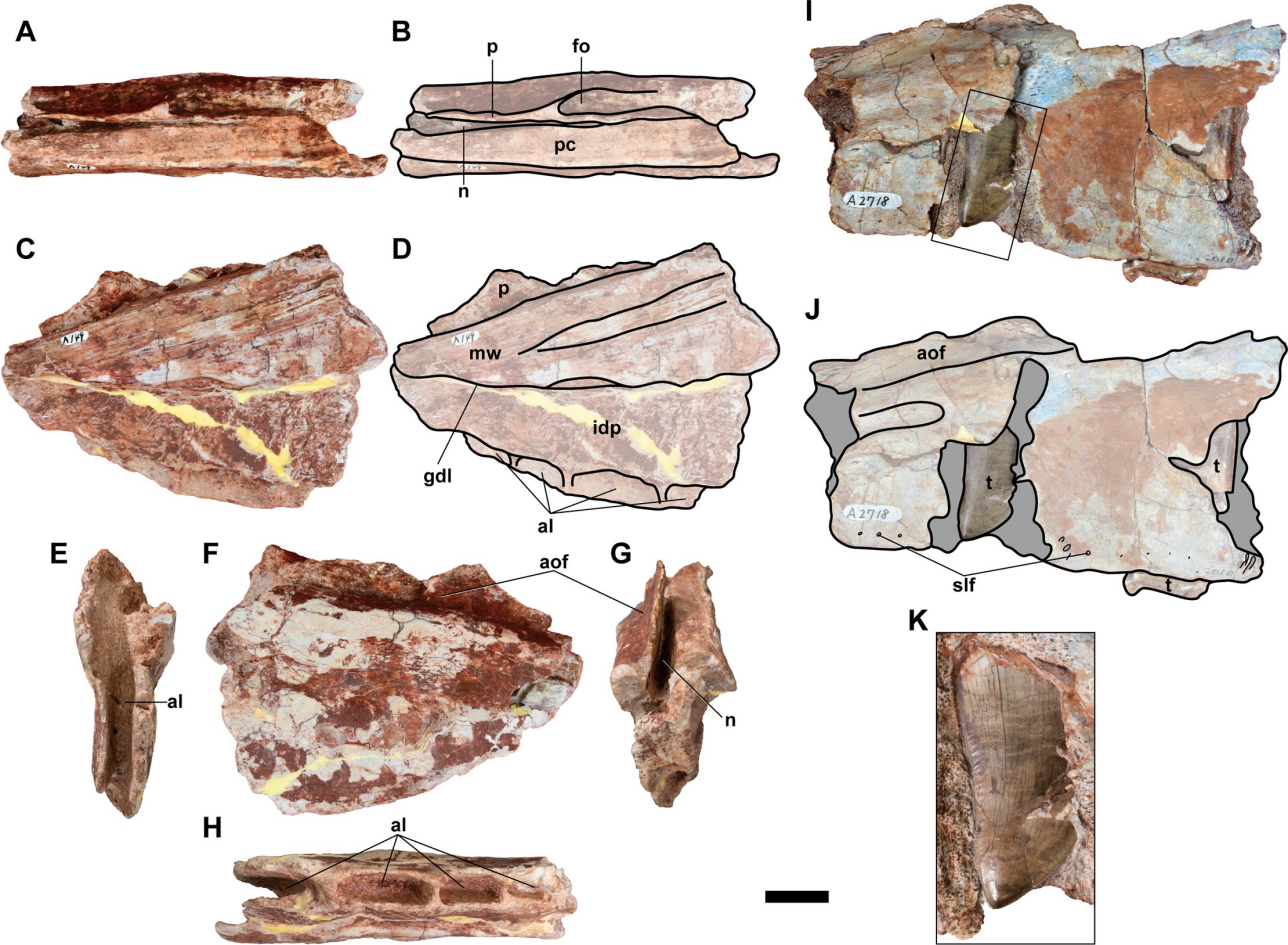
**Left (A–H: NRRU-F01020005) and right (I, J; NRRU-F01020004) maxillae and an *in situ* maxillary tooth (K; NRRU-F010200040) in dorsal (A, B), medial (C, D), anterior (E), lateral (F, I–K), posterior (G) and alveolar (H) views.** The rectangle in I indicates the area magnified in K to show a maxillary tooth. Abbreviations: al, alveolus; aof, antorbital fossa; fo, foramen; gdl, groove for dental lamina; idp, fused interdental plate; mw, medial wall; n, longitudinal notch; p, bony plate; pc, palatal contact; slf, superior labial foramina. Scale bar equals 20 mm for A–J and 10 mm for K.

Partial posteroventral ramus of the right (NRRU-F01020004) and left (NRRU-F01020005) maxillae are preserved (Fig 4). Based on the small size and the absence of any following alveoli, the preserved posteriormost alveolus of the left maxilla should be that of the maxilla itself. The jugal contact is not preserved in both materials. Both taper posteriorly, as in most theropods. Both lateral and medial surfaces are almost smooth.

The lateral surface exhibits the posteroventral part of the antorbital fossa as a depression near the dorsal margin. The ventral rim of the fossa is demarcated by a well-developed ridge, which becomes sharper and step-like posteriorly. The dorsal extent of the antorbital fossa is unknown because of the breakage of a thin, plate-like bone above its ventral rim. Along the ventral margin of the maxilla, the lateral surface is pierced by many small foramina, namely the superior labial foramina for branches of the superior alveolar nerve and maxillary artery.

In medial view, the interdental plates are fused to form a continuous lamina as in most allosauroids, except for *Sinraptor* [14,44,45,48]. However, in NRRU-F01020004, the suture between plates is slightly marked. Most interdental plates are dorsoventrally deeper than anteroposteriorly wide. Above the fused interdental plate, the dorsal half of the medial wall forms a raised area, which is demarcated dorsally by a sharp ridge, and ventrally by the paradental groove, also known as ‘groove for the dental lamina’ (e.g. [49–51]) and ‘nutrient groove’ [52], which separate it from the interdental plates [49]. In NRRU-F01020005, the area is well developed as a striated surface, and the groove composing its ventral margin forms a step-like ridge. A broad, shallow, longitudinal groove is also present in the medial wall in NRRU-F01020004.

In dorsal view, a palatal suture is visible as a narrow shelf dorsal to the medial wall, associated with a longitudinal notch, which is situated lateral to the shelf and deepened posteriorly. This suture is laterally bordered by the bony plate comprising the antorbital fossa. In the left maxilla (NRRU-F01020005), an oval foramen is present laterally adjacent to the bony plate, near the anterior limit of the notch.

The right maxilla (NRRU-F01020004) has four complete alveoli with partial teeth inside and two incomplete alveoli without erupted teeth. Two unerupted teeth are visible in the broken area. Although the left maxilla (NRRU-F01020005) has four alveoli, there are no preserved erupted teeth. All the alveoli, as well as the maxillary teeth, are laterally compressed, anteroposteriorly elongated, and rectangular shaped in ventral view. All the maxillary teeth exhibit the typical shape for carcharodontosaurids, as they are broad in lateral view, slightly recurved, serrated, and transversely thin. Both carinae in all the teeth have denticles, with a density of three denticles per millimeter, as in *Acrocanthosaurus* [53]. Numerous small and non-pronounced marginal enamel wrinkles are present in the distal carinae. In dorsal view of NRRU-F01020004, the second anterior alveolus (the anteriormost one of the complete alveoli) exhibits a replacement tooth adhered to the concave medial side of the root of the erupted tooth.

#### Jugal

**Fig 5:**
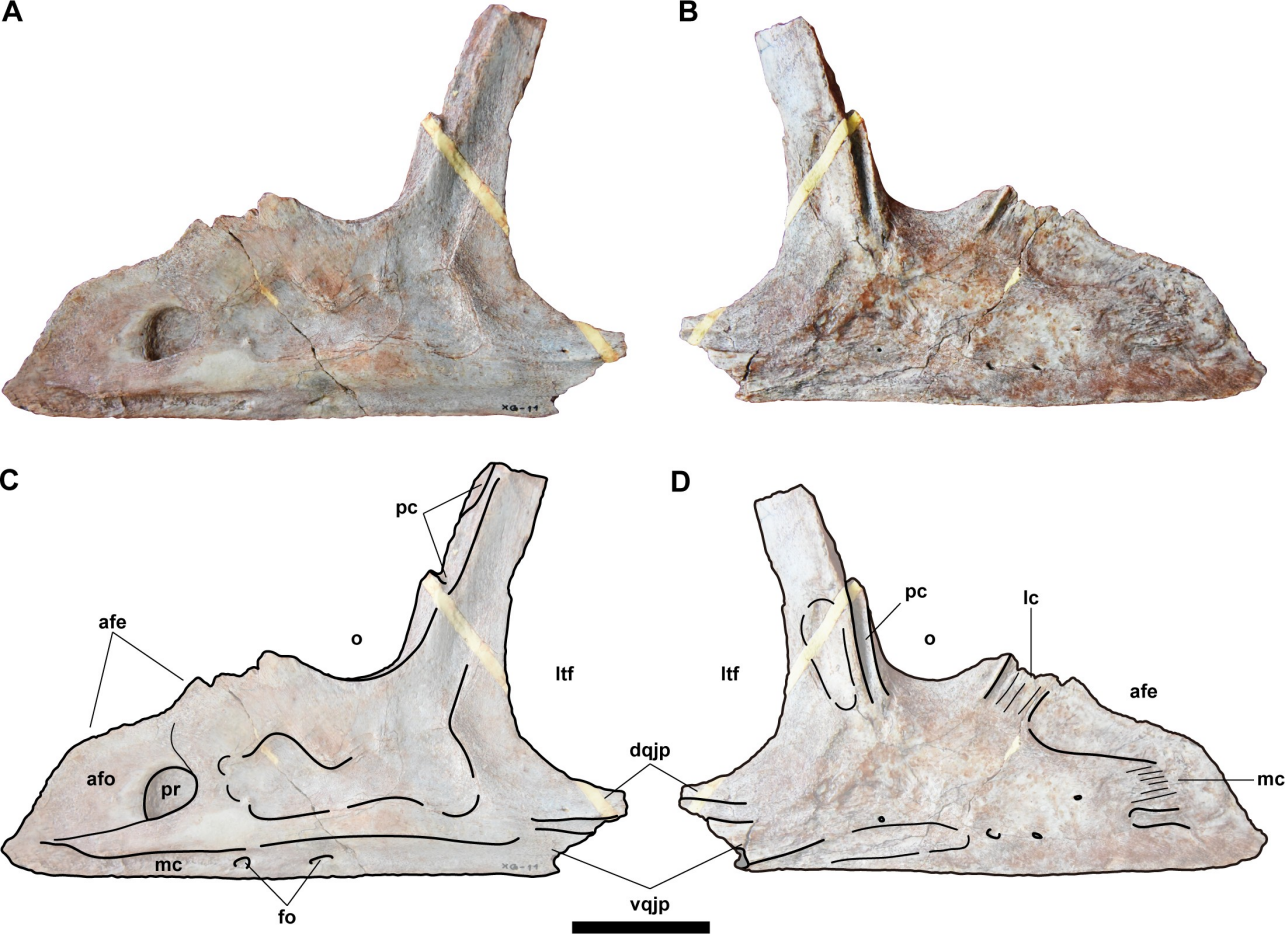
Left jugal (NRRU-F01020006) in lateral and medial views. Abbreviations: afe, antorbital fenestra; afo, antorbital fossa; dqjp, dorsal quadratojugal prong; fo, foramen; lc, lacrimal contact; ltf, lateral temporal fenestra; mc, maxilla contact; o, orbit; pc, postorbital contact; pr, pneumatic recess; vqjp, ventral quadratojugal prong. Scale bar equals 20 mm.

There is a nearly complete left jugal (NRRU-F01020006) that shows a laterally compressed and tripartite shape (Fig 5). The ventral margin of this bone is completely straight in lateral view, unlike in other allosauroids, which present distinct shapes such as undulate [43,44] or slightly bowed ventrally [41,54,55].

In lateral view, there is a concave anterodorsal margin in the anterior process to form the posteroventral corner of the antorbital fenestra. Below the antorbital fenestra, although the antorbital fossa is fairly blunt, there is a circular pneumatic foramen that demarcates the posteroventral corner of the fossa on the lateral surface. This foramen corresponds to the jugal pneumatic recess that is also present in other allosauroids, and it has a similar morphology in *Concavenator* [42]. However, it is divided into two parts in *Sinraptor* [44], and it is much narrower in *Acrocanthosaurus* [41] and *Mapusaurus* [54]. The breaks in the central part of the lateral surface of the jugal indicate the presence of an inner cavity posterior to the foramen, a feature that is also supported by a swelling in the corresponding part of the medial surface. Along the ventral margin in lateral view, there is a narrow groove-like suture for contact with the maxilla in the anterior half of the process. Posterior to this suture, two small foramina and a horizontal ridge are present, the latter running from the anterior end of the groove between quadratojugal prongs. A similar ridge is also seen in *Mapusaurus* and *Tyrannotitan* [55], but it is anterodorsally-oriented toward the ventral margin of the orbit in these species. In medial view, the anterior process has two contact sutures neighboring to the antorbital fenestra, ventrally for the posterior ramus of the maxilla and posteriorly for the descending process of the lacrimal. Both are marked by striations. The posterior part of the anterior process is dorsoventrally tall and has a shallowly concave dorsal margin comprising the ventral margin of the orbit. This is a fairly characteristic condition, given that, in other allosauroids, the orbit excavates the dorsal margin more deeply to reach almost the same level as the ventral margin of the lateral temporal fenestra [41,43,44,56]. A similarly shallow, but strongly narrow concavity in the dorsal margin is also seen in *Monolophosaurus* [57], but it is currently regarded as a basal tetanuran [12].

Posterior to the anterior process, the postorbital process projects dorsally. The anterior margin of the process has a contact suture for the ventral ramus of the postorbital. The suture faces mainly anterolaterally and its ventral half has a dorsally-projecting lamina. In lateral view, the postorbital contact ends at the dorsal margin of the lamina, and thus it is substantially above the ventral rim of the orbit, like in other avetheropods [12,14,47]. In contrast, medial to the lamina, the suture is continuous as a notch and it extends more ventrally up to the base of the postorbital process. A similar notch is also present in *Sinraptor* [44] as the postorbital contact, although it ends high above the ventral margin of the orbit. Posterior to this notch, there is a shallow, dorsoventrally elongate depression.

The posterior process lacks its posterior end and thus it shows the bases of two quadratojugal prongs, which form a tongue-and-groove contact with the anterior ramus of the quadratojugal. The ventral prong is taller than the dorsal prong, which is a similar condition to *Allosaurus* [43], but contrary to most allosauroids [41]. The contact suture for the quadratojugal is also present as the grooves dividing these prongs in both lateral and medial surfaces, which are deeper in the latter. There does not exist an accessory prong between these two prongs, unlike in carcharodontosaurids [41].

#### Surangular

**Fig 6:**
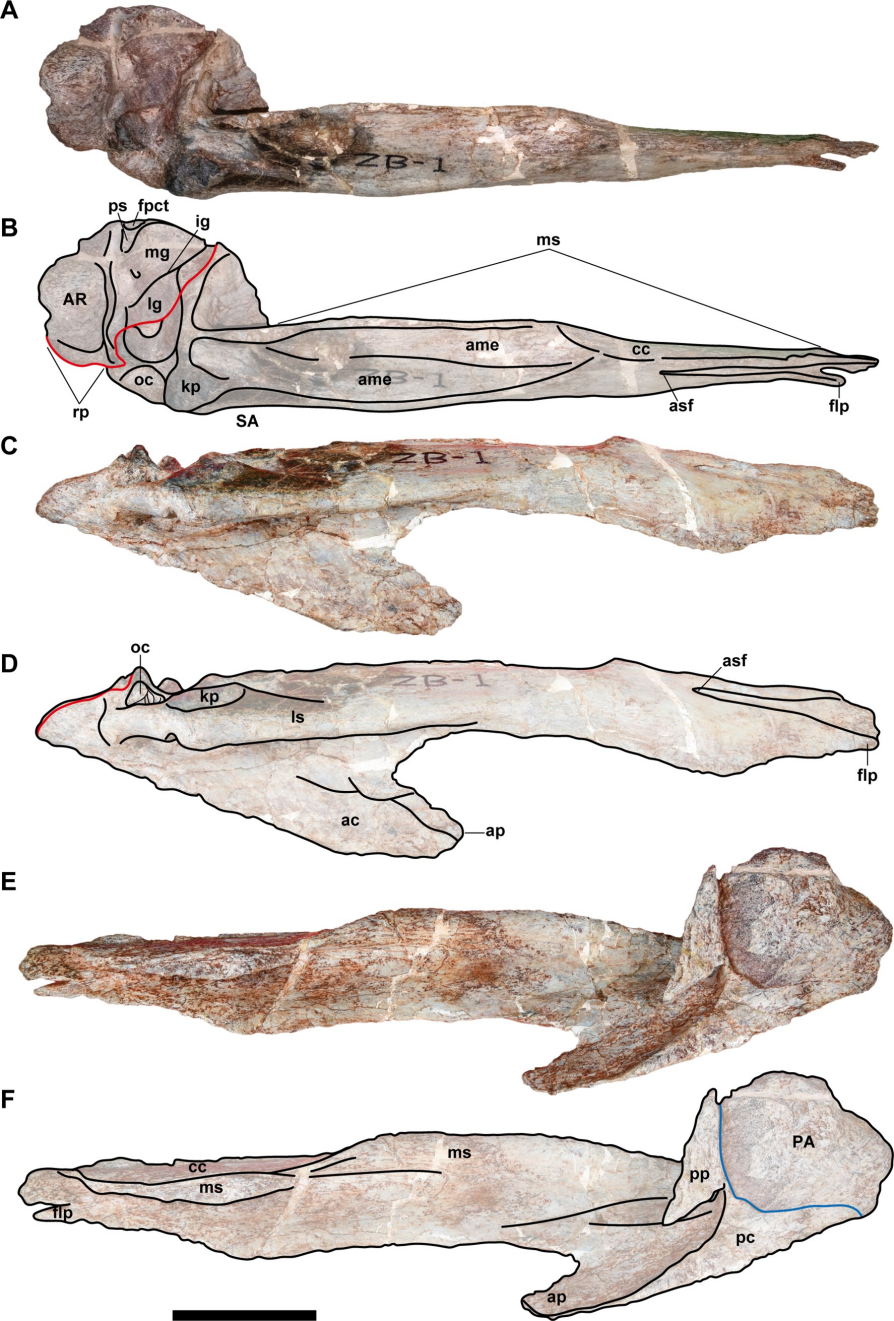
**Posterior part of the right mandible (NRRU-F01020008) in dorsal (A, B), lateral (C, D), and ventromedial (E, F) views.** Red and blue lines indicate the surangular (SA)/articular (AR) and surangular/prearticular (PA) sutures, respectively. Abbreviations: ac, angular contact; ame, *M*. *adductor mandibulae externus* attachment; ap, angular process; asf, anterior surangular foramen; cc, coronoid contact; flp, finger-like process; fpct, foramen posterior chorda tympani; ig, interglenoid ridge; kp, knob-like process; lg, lateral glenoid; mg, medial glenoid; ms, medial shelf; oc, oval concavity; pc, prearticular contact; pp, postadductor process; ps, postglenoid spine; rp, retroarticular process. Scale bar equals 100 mm.

**Fig 7:**
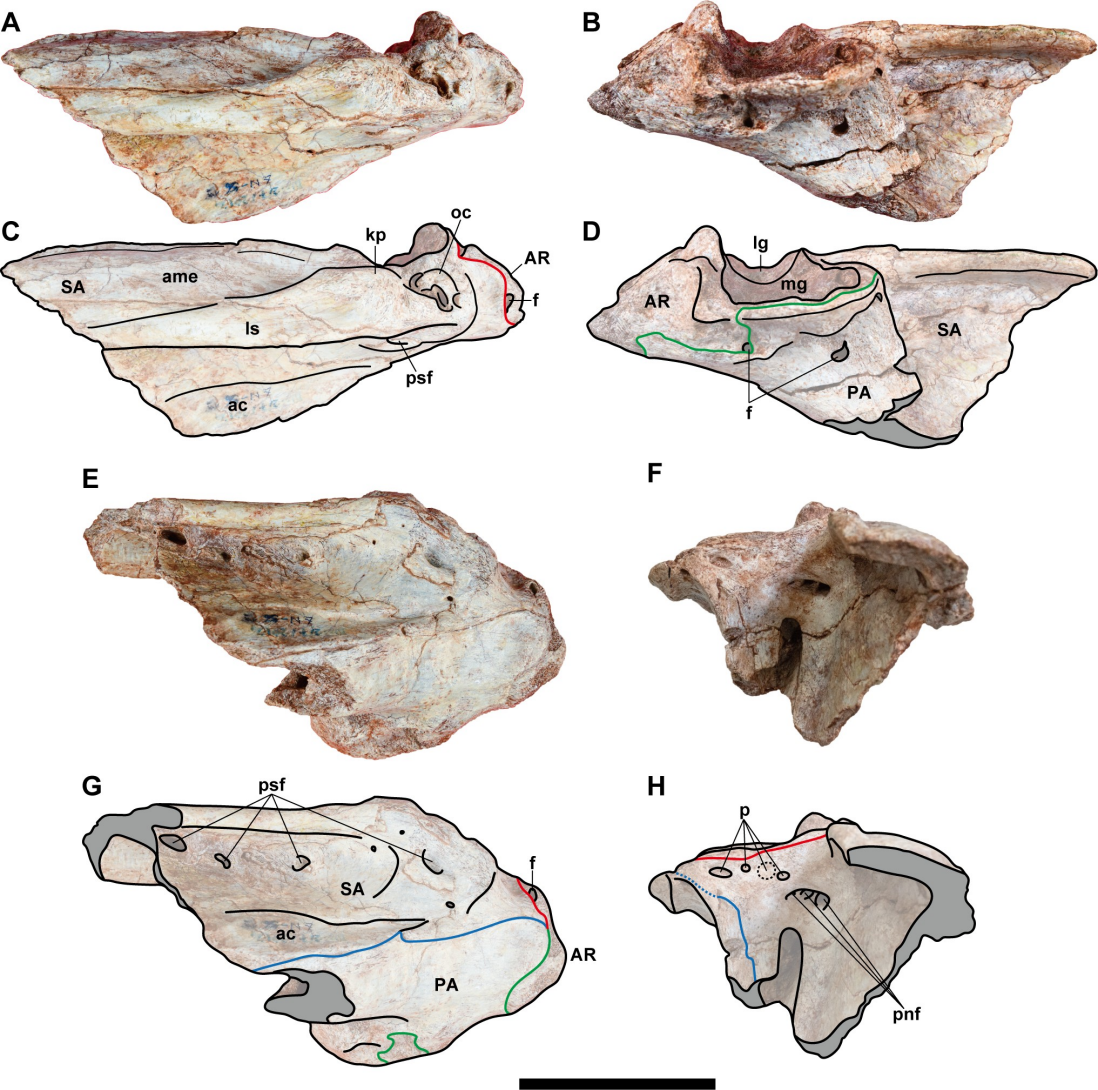
**Posterior part of the left mandible (NRRU-F01020009) in lateral (A, C), medial (B, D), ventral (E, G), and anterior (F, H) views.** Red, blue, and green lines indicate the surangular (SA)/articular (AR), surangular/prearticular (PA), and articular/prearticular sutures, respectively. Abbreviations: ac, angular contact; ame, *M*. *adductor mandibulae externus* attachment; ampsf; anteromedial ends of posterior surangular foramina; f, foramen; kp, knob-like process; lg, lateral glenoid; ls, lateral shelf; mg, medial glenoid; oc, oval concavity; p, pits; pnf, pneumatic foramen; psf, posterior surangular foramen. Scale bar equals 100 mm.

**Fig 8:**
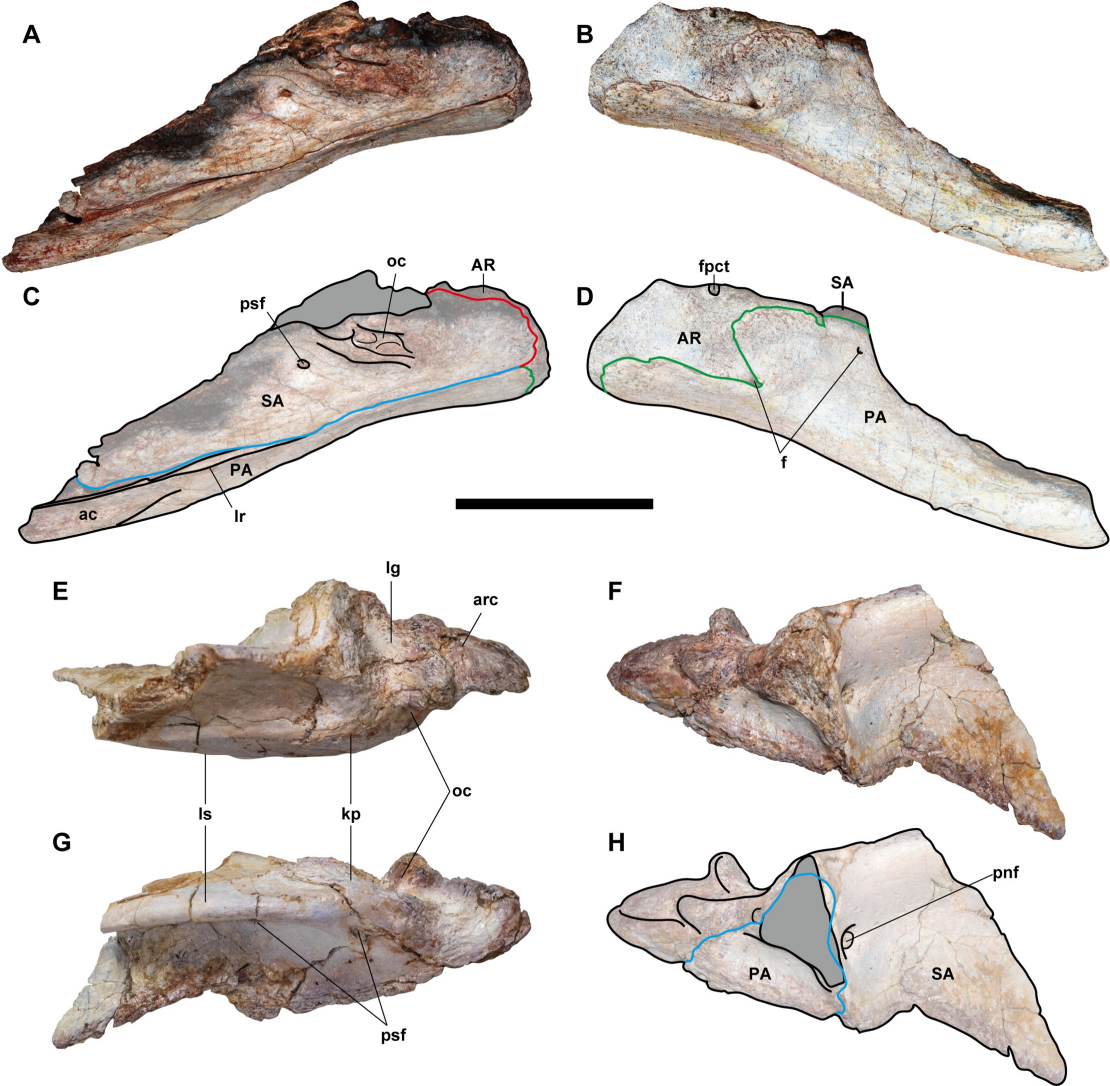
**Posterior parts of the left mandibles, NRRU-F01020007 (A–D) and F01020010 (E–H) in lateral (A, C, G), medial (B, D, F, G), and dorsal (E) views.** Red, blue, and green lines indicate the surangular (SA)/articular (AR), surangular/prearticular (PA), and articular/prearticular sutures, respectively. Abbreviations: ac, angular contact; arc, articular contact; AR, articular; f, foramen; fpct, foramen posterior chorda tympani; kp, knob-like process; lg, lateral glenoid; lr, longitudinal ridge; ls, lateral shelft; oc, oval concavity; PA, prearticular; pnf, pneumatic foramen; psf, posterior surangular foramen; SA, surangular. Scale bar equals 100 mm for A-D and 60 mm for E–H.

Four partial mandibulae are known for this taxon. Each one of NRRU-F01020007–F01020009 is composed of the articular, prearticular and surangular (Figs 6, 7 and 8), whereas NRRU-F01020010 is composed of the prearticular and surangular. The right surangular is virtually complete in NRRU-F01020008, whereas the left surangular preserves only a posterior part in NRRU-F01020007, F01020009, and F01020010.

The anterior surangular foramen is excavated in the posterior end of the anterior one–third of the surangular, and it is continuous anteriorly with a shallow groove. Anteriorly, the ventral margin of the groove is formed by a finger-like process, which, in other theropods, extends laterally over the lateral surface of the dentary. The flat, anteroventrally-projecting flange (angular process) forms a posterior margin of the external mandibular foramen. There is a wide contact surface for the angular along the ventral margin of the process as a slightly depressed surface with blunt oblique striations. This contact is most clearly observed in NRRU-F01020009 with a prominent step-like dorsal margin, and it is posteroventrally delimited by the prearticular. In the lateral surface of the surangular, the lateral shelf is strongly developed slightly above the midline of the posterior half of the bone. It becomes dorsally higher in its posterior part to form a knob-like process situated anterolateral to the lateral glenoid. Medial to the lateral shelf, the medial shelf is also well-developed and located more dorsally. Between these shelves, there is a longitudinal and lateromedially wide depression, on which the *M*. *adductor mandibulae externus* attaches. Slightly anterior to its posterior end, a blunt longitudinal ridge emerges and divides the depression into two parts in NRRU-F01020008 and F01020009. The medial shelf continues further anteriorly and forms a striated, dorsomedial-facing surface as a suture with the coronoid. In this part, the medial shelf also forms a longitudinal cleft that is continuous with an anterodorsally-directed groove bordering the anteroventral margin of the coronoid suture. The dorsal margin of the coronoid suture is marked by a longitudinal ridge. Posteriorly, the medial margin of the medial shelf forms a longitudinal ridge projecting dorsally and is continuous with the lateromedial ridge representing the anterior margin of the glenoid. Ventrally, the surangular forms a hooked process (postadductor process) delimiting the posteromedial margin of the adductor fossa and contacting the prearticular medially. Posterodorsal to the wide adductor fossa in anterior view, there is an elongated fossa with three or four deep foramina separated by septa in NRRU-F01020007 and F01020009. More posteriorly, the surangular forms the lateral part of the glenoid, but it has no contribution to the ridge separating two depressions of the glenoid, unlike in *Sinraptor* [44]. The surangular extends more posteriorly to contribute to the posterolateral end of the retroarticular process. The posterior process of the surangular has a mediodorsally-faced suture for the articular.

Four posterior surangular foramina are preserved beneath the lateral shelf. The most posterior and the biggest one is located anterolateral and ventral to the glenoid, in the same position as that of *Sinraptor* [44]. This foramen penetrates the bone anteromedially and ends on the posteriormost part of its inner surface (adductor fossa) as a part of possible pneumatic foramina. The second foramen is located more anteriorly, anteroventral to the knob-like process of the lateral shelf. It penetrates medially or posteromedially into the bone and conjoins with the first foramen. Anteriorly, there are two additional smaller posterior surangular foramina under the marked lateral shelf. The third (posterior) one penetrates the bone with two directions, namely posteromedially and anteromedially, and only the former pierces into the inner surface of the bone. The fourth (anterior) one penetrates only posteromedially and conjoin with the piercing foramen of the third, just before its end. In theropods, only a single foramen or two foramina have been reported, and the latter condition was proposed as a synapomorphy of Allosauroidea [47].

The posterior end of the lateral shelf is marked by a deep oval concavity excavating the bone medially. Within this concavity, there are five to seven small foramina separated by septa. This feature has not been reported in other allosauroids and thus could be an autapomorphy for this taxon.

In the anterior view of NRRU-F01020009, there are four small pits ventrally along the anterior margin of the glenoid, but they are clearly absent in a smaller specimen (NRRU-F01020008). Lateroventral to these pits, there is a large, mediolaterally-elongated pneumatic foramen on the posteriormost part of the adductor fossa. This foramen penetrates the surangular posteriorly and is separated by dorsoventrally-oriented septa. Laterally, the foramen widens and is continuous with the first and second posterior surangular foramina. Although this foramen is visible in all the available surangular materials, the septa are apparently absent in the smallest specimen (NRRU-F01020010). In NRRU-F01020008, the surangular forms an oval fossa at the middle of the glenoid along the suture line with the articular. Although the glenoid is also well preserved in NRRU-F01020010, the fossa is absent. The absence of these characters in smaller specimens indicate some ontogenetic variations within the surangular.

#### Prearticular

Each of the four posterior mandibles (NRRU-F01020007–F01020010) contains a part of the prearticular, but it is best preserved in NRRU-F01020007 (Fig 8). Each prearticular is preserved in contact with the surangular and, if present, the articular in the medial side of the mandible. Only the thick posterior part of the prearticular is preserved. This posterior part is broadly expanded to underlie the articular to cover its ventral margin and the hooked postadductor process of the surangular. The preserved prearticular is anteriorly extended, forming the ventral margin of the mandible.

In lateral view, anterior to the level of the posterior surangular foramen, the ventral margin of this bone becomes bowed ventrally. On the dorsal margin of the anterior part of this bone, there is a longitudinal ridge projecting laterally. When in articulation with the surangular, the ridge makes a long and narrow groove rather than a normal suture line. Below or anterior to this ridge, the lateral aspect of this bone forms a concave surface, possibly for the contact with the angular. The posterior part of this bone swells medially and becomes Y-shaped in medial view due to a prominent notch at the middle of its posterodorsal margin. There are three open foramina in the medial aspect of the prearticular. The posterior one is formed at the notch of the articular-prearticular suture (see Articular below). The middle one is seen only in NRRU-F01020009 (the largest specimen among the four), and it is situated anterior to the posterior one. The anteriormost one is seen in both NRRU-F01020007 and F01020009, situated at the dorsal margin of the posterior part of the prearticular, just below the anterior margin of the suture with the articular, also described in *Acrocanthosaurus* [41].

#### Articular

Three articulars (NRRU-F01020007–F01020009) are preserved in articulation with the surangular and the prearticular (Figs 6–8). The anterior margin is delimited by a marked ridge of the surangular, as in other Tetanurae [12]. In dorsal view, the articular is as wide as long, with a depressed and strongly wide glenoid region. The glenoid region is not separated in the lateral and medial glenoid fossae by a sharp ridge, like *Allosaurus* [58] or *Sinraptor* [44]. However, this interglenoid ridge is also greatly reduced in *Mapusaurus* and low in *Acorcanthosaurus* [41]. There is a tall and slightly sharp spine, forming the posterior margin of the glenoid. This spine is smaller and less developed than in carcharodontosaurids such as *Acrocanthosaurus* or *Mapusaurus*, but it is similarly developed as in non-carcharodontosaurid allosauroids like *Sinraptor* or *Allosaurus*. Posterior to this spine, there is a laterally-oriented groove, which divides the retroarticular process from the spine. This groove is possibly homologous to that observed in *Tyrannosaurus* [59] and *Murusraptor*, in the latter of which the groove is recognized as the insertion of *M*. *depressor mandibulae* [22]. The semicircular retroarticular process is lateromedially wide and has a concave bowl-like attachment surface facing posterodorsally. A posteriorly-oriented retroarticular process has been proposed as a synapomorphy of Avetheropoda [12,60] or a synapomorphy of Allosauria [12]. However, a posterodorsally inclined retroarticular process is also defined in *Acrocanthosaurus* [41].

In medial view, there are two prominent foramina in NRRU-F01020007, F01020009, and F01020010. The first one is below the approximate middle of the medial glenoid, formed together with the notch of the posterodorsal margin of the prearticular. This foramen is not seen in other allosauroids, so this seems to be a diagnostic feature for this taxon. The second one is the foramen posterior chorda tympani located immediately below the posterior margin of the medial glenoid. This foramen is smaller than those of *Sinraptor* [44] and *Acrocanthosaurus* [41]. The medial surface of the articular presents a projection that envelops this foramen.

#### Cervical vertebra

**Fig 9:**
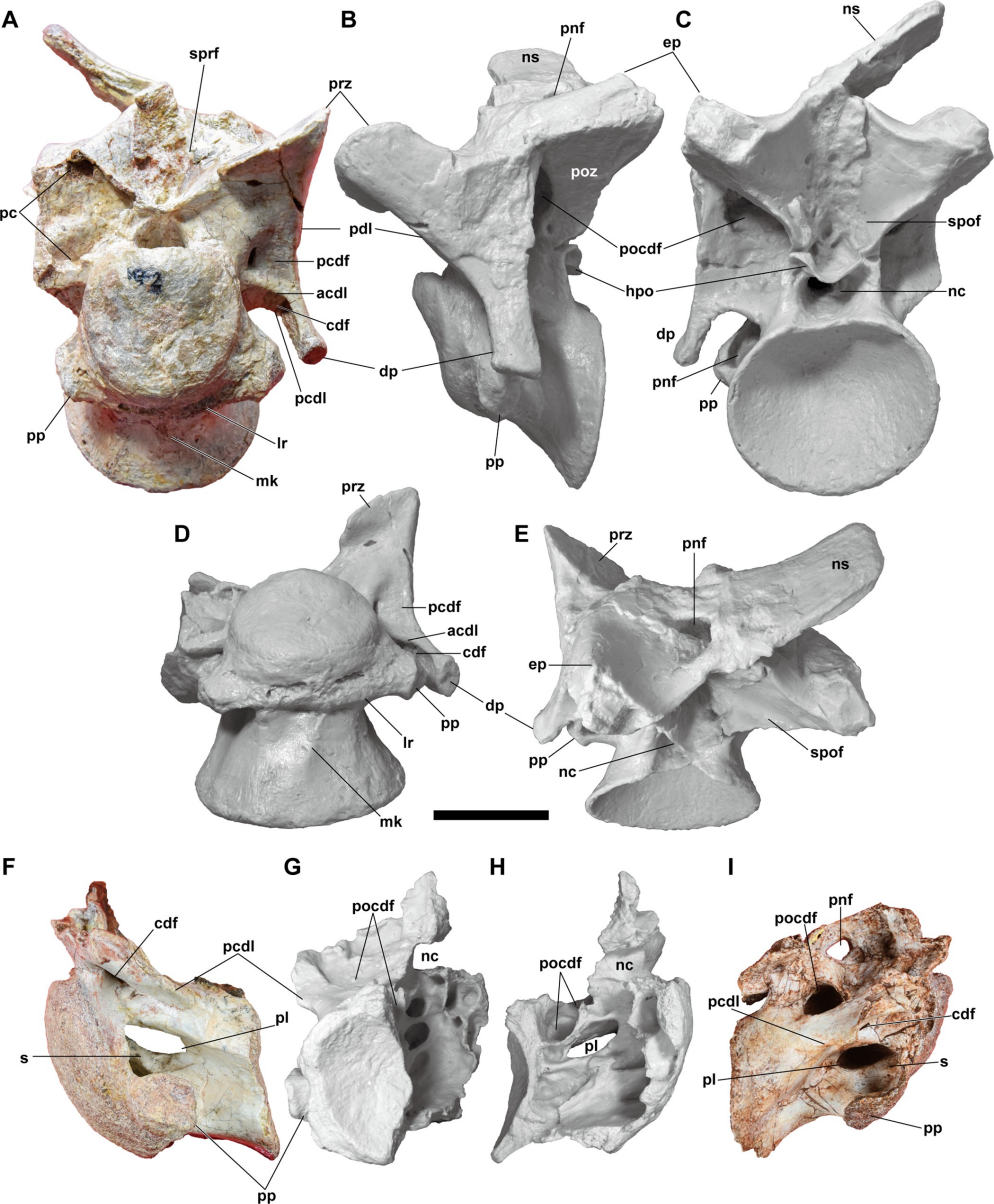
**Cervical vertebrae, NRRU-F01020011 (A–E), F01020012 (F–H), and F01020013 (I) in anterior (A), left lateral (B, F), ventral (C), dorsal (D), posterior (C, G), and right lateral (H, I) views.** Uncolored casts of each specimen are used for B–E, G and H. Abbreviations: acdl, anterior centrodiapophyseal lamina; cdf, centrodiapophyseal fossa; dp, diapophysis; ep, epipophysis; hpo, hyposphene; lr, laterally-oriented ridge; mk, median keel; nc, neural canal; ns, neural spine; pc, pneumatic cavity; pcdf, prezygocentrodiapophyseal fossa; pcdl, posterior centrodiapophyseal lamina; pdl, prezygodiapophyseal lamina; pnf, pneumatic foramen; pocdf, postzygapophyseal centrodiapophyseal fossa; poz, postzygapophysis; pp, parapophysis; prz, prezygapophysis; s, septum; spof, spinopostzygapophyseal fossa; sprf, spinoprezygapophyseal fossa. Scale bar equals 50 mm.

One complete and two incomplete cervical vertebrae are known for this taxon (Fig 9). NRRU-F01020011 is an almost complete cervical vertebra, most likely to be the 3^rd^ in comparison with *Allosaurus* [43], mainly based on a quite short distance between parapophysis and diapophysis and the extent of the anterior projection of the prezygapophysis. NRRU-F01020012 and F01020013 are basically similar to NRRU-F01020011 and have laterally wide centra and relatively flat anterior surfaces, being likely to be the successive cervical vertebrae, namely, in 4^th^ to 6^th^ position. The anteroposteriorly short centra also support that these are not the longer posterior vertebra from the 6^th^ position, like in Neotheropoda [61].

The centrum is opisthocoelous and is invaded bilaterally by a pneumatic foramen on its anterior part. In the 3^rd^ cervical, the anterior articular surface is circular in anterior view and convex, but its ventral half is more flattened than the dorsal half. In the following cervical vertebrae, the anterior surface is laterally elongate, and the opisthocoelic condition is very reduced, like in *Sinraptor* (IVPP V10600), and completely unlike the developed hemispherical condition (“ball and socket”) in derived carcharodontosaurids [55]. The well-excavated posterior surface is offset strongly ventrally relative to the anterior surface and elongated laterally to show an elliptical outline posteriorly, except for its dorsal part where the rim is slightly concave anteriorly. The parapophysis strongly projects laterally from the anteroventral corner of the centrum so that it almost reaches the lateroventral margin of the anterior surface in lateral view. The parapophyseal facet is convex in NRRU-F01020011 and F01020013, whereas it is flat in NRRU-F01020012. In the 3^rd^ cervical, the ventral surface of the centrum is almost flat, except for a laterally-oriented ridge between both parapophyses and a blunt median keel in its anterior two-thirds. The following cervical vertebrae have a completely flat ventral surface without the median keel, whereas the lateral ridge between parapophyses is present as a ventral margin of the anterior surface.

The centrum is camerate due to the division in several huge camerae by septa, as proposed by Britt [62]. This structure is best observed in NRRU-F01020012. In posteromedial view, the lateral wall of the internal cavity of the centrum is penetrated by the pneumatic foramen occupying its posterodorsal margin. The rest of the lateral wall is excavated by several fossae. They are directed anteriorly in the area anterior–anteroventral to the opening and directed posteriorly in the area posterior–posteroventral to the opening. Additionally, a laterally-oriented foramen is present anterodorsal to the opening. Anterior to the large internal cavity, another small cavity, with several laterally-oriented foramina, is present inside the anterior articular surface. Dorsal to the internal centrum cavity, another cavity that opens dorsally toward the infrapostzygapophyseal fossa is present.

The pneumatic foramen is better developed on the left side than on the right side in NRRU-F01020011. The anterior and dorsal margins of the pneumatic foramen are marked by an anterodorsally-bowed lamina which extends from the posteroventral margin of the centrodiapophyseal fossa to the anterodorsal margin of the parapophysis. The pneumatic foramen deepens anteriorly and splits into two, dorsal and ventral, fossae separated by a thick, anterodorsally-directed septum, as in *Aerosteon* [21]. The dorsal fossa of the pneumatic foramen is narrow and deep anteriorly and becomes broader and shallower posteriorly. The anterior part of the dorsal fossa opens medially into the internal chamber of the centrum, at least, in the subsequent cervical vertebrae, whereas the condition is unknown in the 3^rd^ cervical because of the extreme narrowness of the corresponding part. The ventral fossa is almost elliptical, and it is further divided into two parts, the one excavates anteromedially toward the anterior surface and the other anteroventrally into the parapophysis. The posterior and medial margins of the ventral fossa are marked by a posteromedially-bowed lamina that extends from the ventral margin of the septum between the fossae to the posterodorsal margin of the parapophyseal facet. A similar fossa is observed in *Condorraptor* [63] and *Sinraptor* [44], in which the pneumatic foramen is located posterodorsal and dorsomedial to the parapophysis, respectively.

The prezygapophysis is projecting anterodorsally and supported lateroventrally by the prezygodiapophyseal lamina, and medioventrally by a blunt centroprezygapophyseal lamina. Between these laminae, the prezygocentrodiapophyseal fossa is present and has a foramen that is small, laterally narrow, and located near the base of the centerodiapophyseal lamina. The ventral margin of this foramen is also preserved in the broken neural arch of the following cervical vertebrae. This foramen opens into a large pneumatic cavity within the prezygapohysis that is visible in the broken right side. The tab-like prezygapophyseal facet faces dorsally but somewhat inclines anteromedially as in *Sinraptor* [44], and unlike in *Allosaurus* [43]. The anterior margins of the left and right prezygapophyseal facets are medially continuous as thin laminae and they meet at their medial ends to form the V-shaped base of the spinoprezygapophyseal fossa. This fossa deepens medially, and the deepest part has a rugosity that indicates an attachment of the interspinal ligament.

The diapophysis is projected strongly ventrally and slightly posterolaterally, as in the 3^rd^ cervical of *Allosaurus* [43], and it has a ventrally-facing flat facet. In lateral view, the anterior and posterior margins are demarcated by the anterodorsally-inclined prezygodiapophyseal lamina and the subvertically oriented postzygodiapophyseal lamina, both of which make the diapophysis being subtriangular, as seen in some carcharodontosaurians [13]. The centrodiapophyseal laminae are inclined anteriorly with the anterior lamina directed dorsally and the posterior lamina directed anterodorsally in their basal part, as in the anterior cervical vertebrae of other allosauroids. The posterior centrodiapophyseal lamina is thicker and longer than the anterior. The centrodiapophyseal fossa is present between these laminae with several deeper fossae inside. At least in the medial part, the fossa is larger on the right side than on the left, contrary to the condition of the pneumatic foramen. The medial part is wide and has a circular outline on the right side, whereas it is narrow and triangular and is ventrally bordered by the dorsal rim of the pneumatic foramen on the left side. The lateral part of the centrodiapophyseal fossa extends until the ventral margin of the diapophyseal facet.

The postzygapophyses projects laterally and slightly posteriorly from the base of the neural spine. The postzyagpophyseal facet faces lateroventrally and slightly posteriorly. The facet is elongated ventromedially to the level slightly above the neural canal, as in the anterior cervical vertebrae of other allosauroids. The ventral margin of the facet is supported by a short, vertical lamina demarcating the lateral margin of the neural canal. Posterior margins of the left and right facets ventrally emerge as fairly thin laminae and meet at their medioventral ends to form a V-shaped hyposphene that delimits the base of the spinopostzygapophyseal fossa. The posterodorsal margins of both postzygapophyses also demarcate the dorsal margin of the spinopostzygapophyseal fossa, except for its mediodorsal part, in which the fossa is continuous as a groove on the posterior surface of the neural spine. This fossa medially deepens and has a rugosity indicating an attachment of the interspinal ligament as in the spinoprezygapophyseal fossa. The postzygocentrodiapophyseal fossa is present between the postzygodiapophyseal lamina and the anteroventral margin of the postzygapophysis, and above the posterior centrodiapophyseal lamina. This fossa is the largest pneumatic fossa in the cervical vertebra. The fossa invades the neural arch dorsomedially and has a deeper dorsomedial part distinguished by a step-like lateroventral margin, as in *Sinraptor* (IVPP V10600). The epipophysis is present as a mound-like swelling on the dorsal surface of the postzygapophysis, unlike a large posteriorly-oriented projection of *Sinraptor* [44], *Concavenator* [64], *Tyrannotitan* [55], *Mapusaurus* (MCF-PVPH 108.90), and other theropods such as ceratosaurids [12].

The neural spine is laterally thin and plate-like and seems to be projected purely dorsally. The anterior and posterior surfaces are narrow, but they have longitudinal grooves continuous to the spinopre- and spinopostzygapophyseal fossae, respectively. The base of the neural spine is invaded by pneumatic foramina bilaterally in NRRU-F01020011 and F01020013 and the left side is larger than the right, as in the pneumatic foramen in NRRU-F01020011.

#### Posterior dorsal vertebra

**Fig 10:**
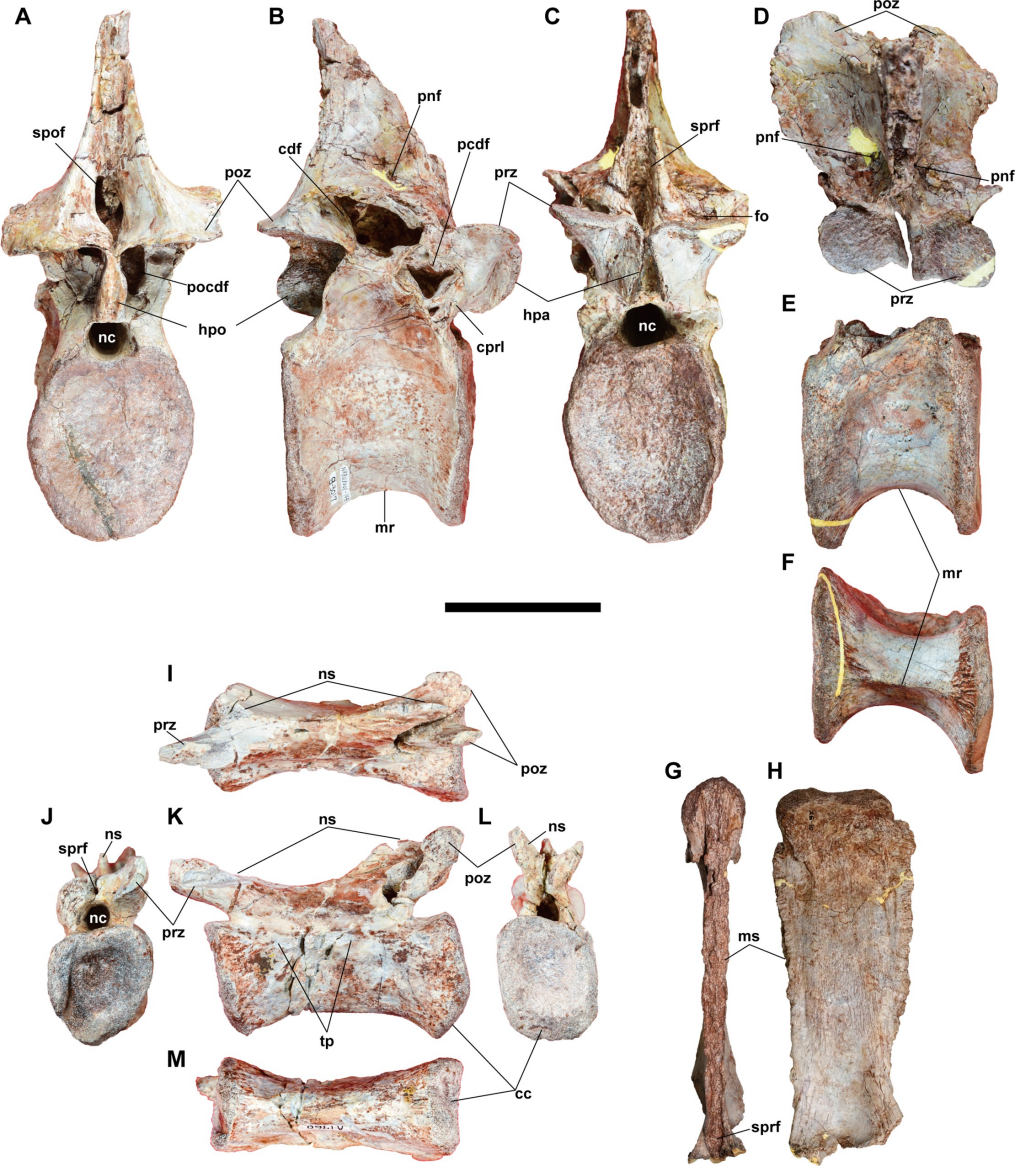
**Dorsal vertebrae NRRU-F01020014 (A–D), F01020015 (E, F), and F01020016 (G, H) and a caudal vertebra NRRU-F01020017 (I–M) in posterior (A, F, L), right lateral (B, E), anterior (C, G, J), left lateral (H, K), dorsal (I), and ventral (F) views.** Abbreviations: cc, chevron contact; cdf; centrodiapophyseal fossa; cprl, centroprezygapophyseal lamina; fo, foramen; hpa, hypantrum; hpo, hyposphene; mr, median ridge; ms, metaplastic scars; nc, neural canal; ns, neural spine; pcdf, prezygocentrodiapophyseal fossa; pocdf, postzygocentrodiapophyseal fossa; pnf, pneumatic foramen; poz, postzygapophysis; prz, prezygapophysis; spof, spinopostzygapophyseal fossa; sprf, spinoprezygapophyseal fossa; tp, transverse process. Scale bar equals 100 mm for A–H and 60 mm for I–M.

Three posterior dorsal vertebrae are known for this taxon (Fig 10). NRRU-F01020015 and F01020016 only preserve the centrum and the neural spine, respectively. NRRU-F01020014 is more complete than the other two, lacking the anterodorsal part of the neural spine, transverse processes, and parapophyses. Both NRRU-F01020014 and F01020015 are slightly distorted to offset the posterior surface to the left side relative to the anterior surface, but they are still parallel to each other. Compared with *Allosaurus* [43], the absence of pneumatic foramina, the strong constriction of the centrum, and the absence of a parapophysis in the mid-height of the centrum indicate that NRRU-F01020014 and F01020015 are posterior vertebrae from the 7^th^. Judging from the dorsal margin perpendicular to both anterior and posterior margins, NRRU-F01020016 is a thin, plate-like spine projecting almost completely dorsally like in NRRU-F01020014 and the posterior dorsal vertebrae from the 8^th^ of *Allosaurus* and *Sinraptor*.

The centrum is higher than transversally wide in both anterior and posterior views. In NRRU-F01020015, the centrum is platycoelous with flat anterior and slightly concave posterior surfaces. In contrast, the anterior surface is slightly concave, and thus, the centrum is amphicoelous in NRRU-F01020014. In lateral view, the centrum is as high as long in NRRU-F01020014 and F01020015. Both the anterior and posterior surfaces exhibit a dorsoventrally-elongated elliptical outline, but their dorsal margin is excavated by the ventral margin of the neural canal. In both materials, the posterior surface is facing slightly more ventrally than the anterior. The mid-section of the centrum is strongly constricted, in such a way that it is hourglass-shaped (constriction *sensu* Carrano et al. [12]). This constriction, which has been previously proposed as a synapomorphy of Metriacanthosauridae [61], is an unambiguous synapomorphy of Allosauroidea present only in their posterior dorsal vertebrae [12]. The ventral surface has a sharp midline ridge, as in 6^th^ and 7^th^ dorsal vertebrae of *Neovenator*, although it is not well developed as the ventral keel (hypapophyses) of the anterior elements in other theropods. The centrum lacks pneumatic foramina on both lateral surfaces, unlike carcharodontosaruids and *Neovenator* [65], which have pneumatic foramina in all dorsal vertebrae, including the posterior.

The prezygapophysis projects anteriorly from the base of the neural spine and its facet faces dorsally. The margin between the neural spine and the prezygapophysis is marked by a small foramen. Both prezygapophyses are closely located to each other to form a narrow cleft, the hypantrum. The hypantrum emerges beneath the prezygapophyses in anterior view and become slightly broader as it approaches its ventral margin. The prezygocentrodiapophyseal fossa, which is best preserved on the right side of NRRU-F01020014, is deeply excavated, as in other avetheropods [12]. The outline of the fossa is marked dorsally by a horizontally-oriented prezygodiapophyseal lamina and by an anterodorsally-directed centroprezygapophyseal lamina. The posterodorsal and posteroventral margins of this fossa are marked by thin broken walls of the bone indicating the presence of thin laminae. A short posterodorsally-oriented lamina extending from the anterodorsal margin of the lateral surface of the centrum supports the parapophysis, as in the 10^th^ of *Sinraptor* [44].

Posterodorsal to the prezygocentrodiapophyseal fossa, there is another large pneumatic foramen composing the centrodiapophyseal fossa at the base of the transverse process. This fossa is surrounded by a thin broken section, except for its ventral margin that is marked by a posteriorly oriented lamina. The deepest part of the centrodiapophyseal fossa is continuous to the inner cavity of the neural arch, as in the prezygocentrodipaophyseal fossa.

The postzygapophyses project slightly posteriorly from the base of the neural spine, and their facet faces ventrally. The facet lacks flange-like lateral extensions unlike that of neovenatorids [14]. The posterior margins of postzygapophyses meet at their medial ends to form the ventral border of the spinopostzygapophyseal fossa. Ventral to this junction, the sheet-like hyposphene extends ventrally with parallel lateral edges, unlike the ventrolaterally divergent triangular shape in other theropods. This parallel condition has been defined as a synapomorphy of Carcharodontosauridae [12]. However, other allosauroids also have this parallel hyposphene, such as some vertebrae of *Allosaurus* (UMNH-VP 9029, 9060, 10111; BYU 17532, 9063), *Neovenator* (MIWG 6348; BMNH R10001), and *Murusraptor* (MCF-PVPH-411). The hyposphene lacks a step-like ridge running posterodorsally from the dorsal border of the neural canal to the posterior edge of the postzygapophyses, as in some megalosauroids and megaraptorans [12,63].

Between the articular surfaces of postzygapophysis and hyposphene, the postzygocentrodiapophyseal fossa is excavated and opens into a huge cavity inside the neural arch, which is also continuous with other major fossae in the neural arch. The lateral margin of the postzygocentrodiapophyseal fossa forms a thin lamina that composes the ventral part of the posterior centrodiapophyseal lamina.

The neural spine is plate-like, and it is higher than anteroposteriorly wide in lateral view. In dorsal view, the spine is a transversally thin sheet, unlike the I-beam cross-sectioned neurapophysis of the derived carcharodontosaurids [66]. In NRRU-F01020014, the spine projects almost perpendicularly to the anteroposterior axis of the centrum as in the 9^th^ dorsal vertebra of *Sinraptor* [44]. Ventrally, perpendicular flanges on the lateral margins emerge on the anterior and posterior surfaces of the neural spine. The spinoprezygapophyseal and spinopostzygapophyseal fossae are present between these flanges. A pronounced roughness representing interspinous ligament metaplastic scars is present within these fossae, and it is dorsally continuous to occupy the anterior and posterior surfaces of the neural spine. The ventromedial part of the spinopostzygapophyseal fossa has a large opening into the huge cavity inside the neural arch. The dorsal end of the spine swells laterally as in *Allosaurus*, but is more strongly developed laterally to form small overhangs at its anteroventral and posteroventral margins. The base of the spine is penetrated by a pair of small foramina bilaterally, which are situated about one-third from the anterior margin of the spine. In dorsal view, within the broken area of the neural spine, a wide anterocentral lumen is observed, although it is not as anteroposteriorly long as the one described in *Aerosteon* [21].

#### Middle caudal vertebra

A caudal vertebra (NRRU-F01020017) lacking the right prezygapophysis and both transverse processes is known for this taxon (Fig 10). The presence of an anterior and posterior ramus of the neural spine and its height, as well as the presence of a small transverse process, indicate that this vertebra could be around the 25^th^ middle caudal vertebra, comparing with *Allosaurus* [43]. The spool-shaped centrum has a triangular anterior surface with a ventral apex and a sub-rectangular posterior surface. Both surfaces are as wide as high, and slightly concave. The ventral portion of the posterior surface is beveled strongly anteriorly for articulation with the chevron. Ventrally, the centrum is pinched as a blunt longitudinal keel, and it slightly widens and flattens near the chevron articulation.

The neural arch is dorsoventrally thin and anteroposteriorly elongate. The prezygapophysis is projected further anteriorly than the anterior surface of the centrum, and its facet is dorsomedially oriented. The transverse processes are incomplete, but they are situated below the level of the dorsal margin of the centrum and they are anteroposteriorly narrow, as in 22^nd^–25^th^ caudal vertebrae of *Allosaurus*. The postzygapophyses are posteriorly projected from the base of the neural spine, but do not extend the posterior end of the centrum. The articular facet is oriented laterally and slightly ventrally. The neural spine is split into anterior and posterior processes, the last one is not dorsally high. The spinoprezygapophyseal fossa is reduced to a small circular recess that penetrates the anterior surface of the base of the neural spine as in the middle caudal vertebrae of *Neovenator* [45]. The neural spine is present as an anteroposteriorly long and laterally narrow sheet. The dorsal margin of the neural spine is concave at its middle part in lateral view. Anterior and posterior to this concavity, the broken bases of the anterior spur and the rod-like process are present, as in the mid-caudal vertebrae of other allosauroids [67].

#### Manual ungual

**Fig 11:**
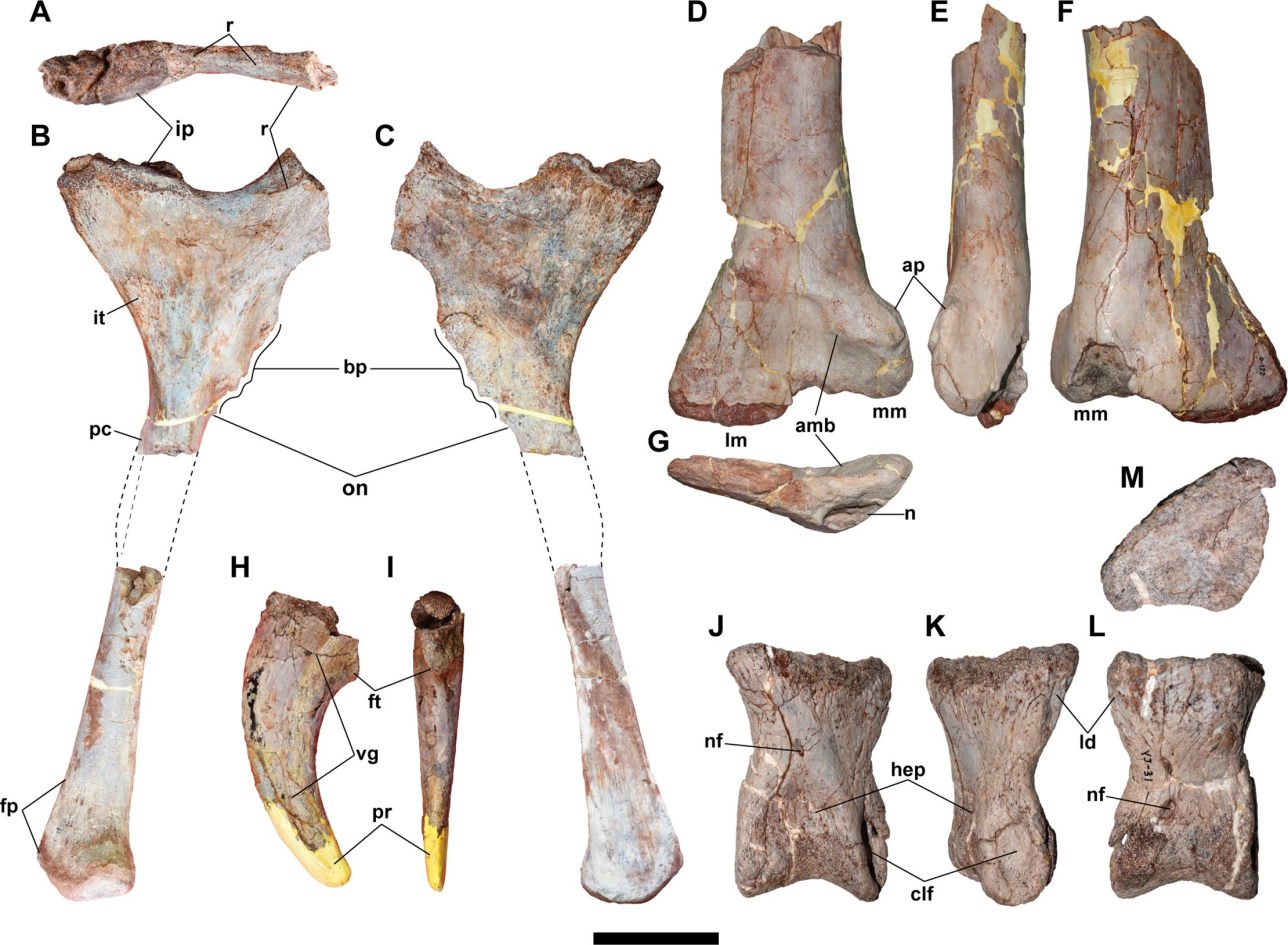
Appendicular skeleton of the new taxon. **Right ischium (NRRU-F01020019 and F01020020) in proximal (A), lateral (B), and medial (C) views, right tibia (NRRU-F01020021) in anterior (D), medial (E), and posterior (F) views, manual ungual (NRRU-F01020018) in lateral (H) and palmar (I) views, and left pedal phalanx IV-1 (NRRU-F01020022) in dorsal (J), lateral (K), plantar (L), and proximal (M) views.** Abbreviations: amb, anteromedial buttress; ap, apex of the medial malleolus; bp, broken end of the obturator process; clf, collateral ligament fossa; fp, flange-like projection; ft, flexor tubercle; hep, hyperextensor pit; ip, iliac peduncle; it, ischial tuberosity; ld, longitudinal depression; lm, lateral malleolus; mm, medial malleolus; n, notch; nf, nutrient foramen; on, obturator notch; pc, posterior crest; pr, plaster reconstruction; r, ridge; vg, vascular groove. Scale bar equals 100 mm for A–G and 50 mm for H–M.

NRRU-F01020018 is a manual ungual lacking only its proximal and distal ends (Fig 11). The preserved proximoventral margin exhibits an anterior end of the flexor tubercle. As seen in megaraptorids, the ungual is mediolaterally thin and dorsoventrally low even in the part near the proximal end. The ungual is not strongly recurved, unlike those of some allosauroids such as *Allosaurus* (UMNH-VP 9718), *Concavenator* (MCCM-LH 6666), *Fukuiraptor* (FPDM-V43), and *Saurophaganax* (OMNH 780). Some derived carcharodontosaurids, such as *Mapusaurus* (MCF-PVPH 108.14) and *Acrocanthosaurus* (NCSM 14345), have less recurved unguals, and they are transversally broad, unlike in *Siamraptor*. Both medial and lateral surfaces have a single vascular groove along the ventral margin in a fixed distance.

#### Ischium

A proximal portion of the right ischium (NRRU-F01020020) is preserved, lacking most of the shaft and the articulation for the pubis (Fig 11). In the proximal portion, the lateral surface is flat or slightly convex, whereas the medial surface is shallowly concave. The iliac peduncle flares laterally at its dorsal margin. In proximal view, although the posteromedial part of the contact surface for the ilium is rugose because of the erosion, the remaining part exhibits a flat surface, like in other basal allosauroids (e.g., *Allosaurus* and *Sinraptor*) and unlike the peg-and-socket articulation in abelisauroids, a neovenatorid *Siats meekerorum* [68] and carcharodontosaurids [12,69]. Anterior to the iliac contact, there is a blunt ridge oriented anterolaterally, which makes two shallow concavities between the iliac and pubic peduncles in the acetabular surface. In lateral view, the base of the pubic peduncle also has a blunt ridge oriented horizontally, just above the midline of the peduncle. The posterior margin of the iliac peduncle is almost straight and directed anteroventrally, so that the posteriorly-directed flange is absent like in basal allosauroids and *Neovenator*, unlike *Allosaurus* and carcharodontosaurids [11]. Between the iliac peduncle and the shaft of the bone, there is a rugose swelling on the lateral surface, the ischial tuberosity, which indicates the attachment of M. flexor tibialis internus [70,71]. The ischial tuberosity is reduced to a slight rugosity in allosauroids such as *Neovenator*, *Allosaurus*, *Concavenator*, and *Acrocanthosaurus*, among other theropods [45,70,72]. In *Siamraptor* this scar is slightly more marked, even more than in *Siamotyrannus* (PW9-1). Although the obturator process is also broken at its base, the proximal and distal obturator notches are present, separating the process from the pubic peduncle and the shaft, respectively. At the level of the ventral end of the obturator process, the posterior margin of the proximal shaft exhibits a beginning of a distinct crest, which seems to be as those observed in *Sinraptor* and other metriacanthosaurids [44] but not in *Allosaurus* (BYU 12906, 16942; UMNH-VP 9505, 20726) and *Acrocanthosaurus* (NCSM 14345).

The distal part of the ischium (NRRU-F01020019), which probably belongs to the same bone as the proximal part (NRRU-F01020020), has convex lateral and flat medial surfaces (Fig 11). In lateral view, a blunt longitudinal ridge runs along the midline of the shaft. Distally, the posterior margin of the shaft forms a thin, flange-like projection, which makes the area posterior to the longitudinal ridge slightly broader than the anterior area. The distal end is only slightly flared anteroposteriorly, as in *Allosaurus*. The medial surface has well-developed striations except for its proximal part, in which the surface becomes smooth and swells medially. There is no sign of fusion with the opposite ischium.

#### Tibia

Only the distal part of the tibia (NRRU-F01020021) is preserved (Fig 11). The shaft is almost straight, mediolaterally wider than anteroposteriorly thick, and mostly flat anteriorly and convex posteriorly, in the preserved distal portion. Distally, the shaft is expanded transversely. On the anterior aspect of this expansion, there is a contact for the ascending process of the astragalus that is represented by a triangular, slightly concave surface. Its proximomedial margin (suprastragalar buttress) is represented by an oblique blunt ridge, as seen in *Acrocanthosaurus*, *Mapusaurus*, and *Neovenator*, unlike the step-like ridge seen in more basal allosauroids such as *Allosaurus* [43] and metriacanthosaurids [44,73]. The lateral malleolus is larger and projecting farther distally than the medial malleolus, as in *Acrocanthosaurus* [66], *Mapusaurus* [54], and *Nevenator* [45]. The lateral margin of the lateral malleolus is straight and directed distolaterally as in *Australovenator* [74] and *Mapusaurus* [54], unlike the rounded one seen in other allosauroids [12]. The medial malleolus projects medially at the same degree as *Allosaurus* [43], *Neovenator* [45], and *Concavenator* [72], unlike the more medially projecting one in *Acrocanthosaurus* [66]. The medial margin of the medial malleolus is rounded and has a blunt apex at its middle part as in *Allosaurus* [43] and *Concavenator* [72]. This apex is more prominent in *Acrocanthosaurus* [66], although is absent in *Neovenator* [45] and *Sinraptor* [44]. Posteriorly, the medial malleolus has a flat, posteromedially-faced surface. On the posterodistal margin, there is a notch just medial to its midline, as seen in metriachanthosaurids [44,73].

#### Pedal phalanx IV-1

NRRU-F01020022 is somewhat deformed (Fig 11). The proximal articular surface is a single shallow concavity, indicating the articulation with a ball-shaped distal surface of the metatarsal IV as in other theropods. In dorsal view, there is a depression just proximal to the distal articular surface (hyperextensor pit). More proximal to that, a small nutrient foramen penetrates the bone distoventrally. The medial collateral ligament fossa is much deeper and more ventrally situated than the lateral one. In ventral view, there is a narrow, longitudinal depression at its proximolateral end, as well as a nutrient foramen just proximal to the distal articular surface.

## Discussion

### Phylogenetic analysis

Several phylogenetic matrices have been used in order to study the phylogenetic relationships of theropod in recent years (e.g. [1,11,12,14,47,67,75–78]). Focusing on the basal Tetanurae, two data matrices are widely recognized as the main ones on the discussion of the alternative distributions of allosauroids, related to the intense debate about the position of Megaraptora. Therefore, we have tested the datasets proposed by Carrano et al. [12] and Porfiri et al. [13] (modified from Novas et al. [76]) in order to establish the phylogenetic relationships of *Siamraptor*. The data matrix of Carrano et al. [12] consists of 361 characters and 62 taxa, and the one of Porfiri et al. [13] is performed by 284 characters and 46 taxa. There are no modifications of these matrices, except for one character in Carrano et al. [12] (ch.132), in which one additional state of character has been added to include the condition in *Siamraptor* (See S1 Appendix).

The matrices were managed using Mesquite 3.01 [79]. Subsequently, these matrices were imported into TNT 1.5 [80] in order to perform a heuristic tree search and find the most parsimonious trees (MPTs). The heuristic tree search used the New Technology algorithms: sectorial searches, ratchet, tree-drifting, and tree fusing, using the default settings for all of them. These algorithms were applied to new searched trees using the driven search with a stabilization of the consensus twice with a factor of 25. Subsequently, the results were exposed to the branch-swapping algorithm of tree bisection reconnection (TBR). The character was ordered only in Porfiri et al. [13], as indicated in their methodology. The MPTs found from both iterations were examined under the strict consensus, and the consistency index (CI) and retention index (RI) were obtained using the “stats.run” script of TNT. The branch support was tested using the methodology proposed by Goloboff et al. [81] to calculate Bremer Support values and the resampling methods bootstrap and jackknife (under default settings). Additionally, in order to find “wildcards” taxa, a reduced consensus tree was performed using the agreement subtrees method. In addition, pruned trees were carried out, calculating pruning up to five taxa.

**Fig 12:**
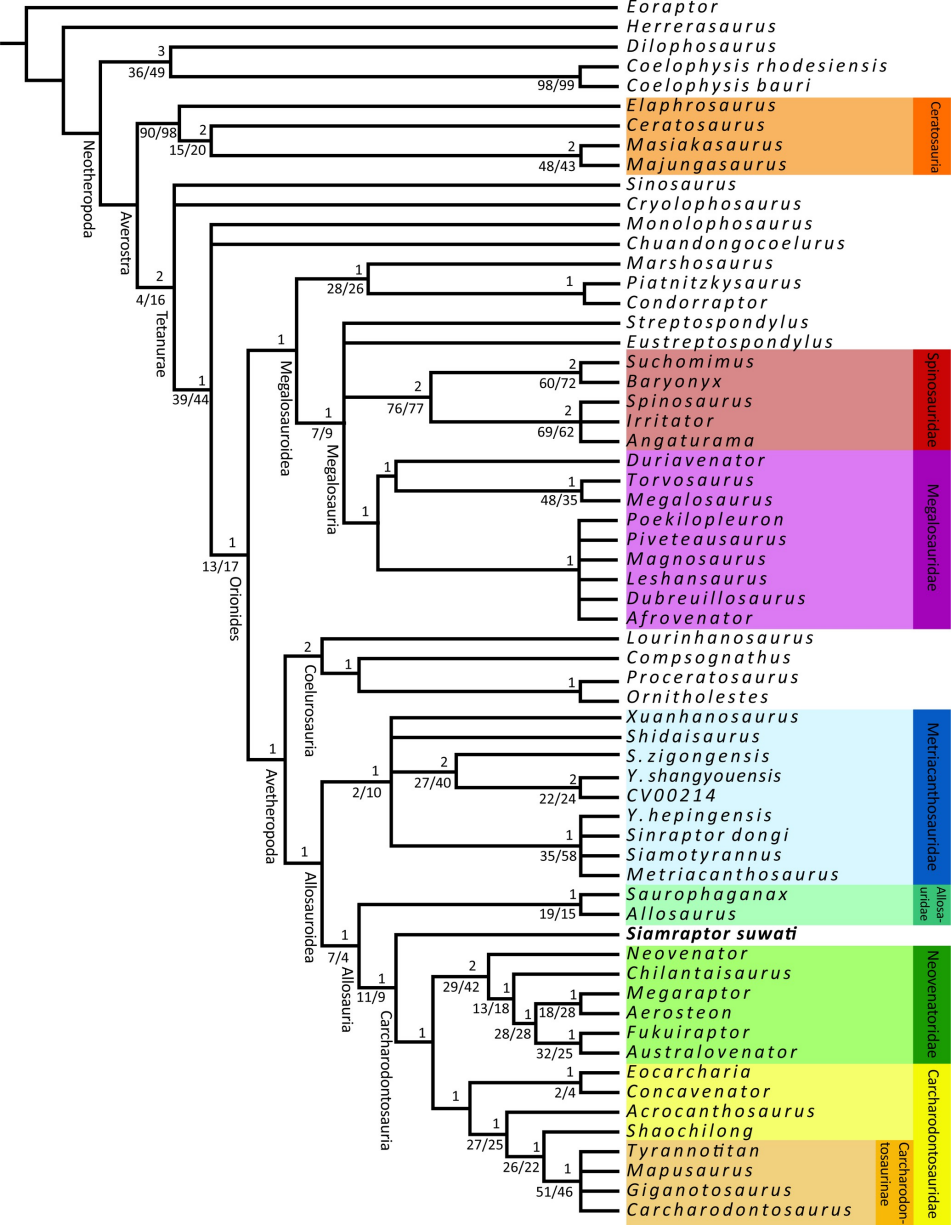
Strict consensus of the phylogenetic analysis based on Carrano et al. **[12] matrix.** The numbers at the top of the nodes indicate Bremmer support, those at the bottom are bootstraps and jackknife branch support, respectively.

The heuristic tree search using the data matrix based on Carrano et al. [12] produced 972 MPTs of 1016 steps, with a consistency index of 0.416 and a retention index of 0.695 (Fig 12). *Siamraptor* is nested within Allosauria in the strict consensus as a more derived position than Allosauridae (*Allosaurus + Saurophaganax* sensu Carrano et al. [12]) and as the most basal taxon of Carcharodontosauria (the most inclusive clade including Neovenatoridae and Carcharodontosauridae and excluding Allosauridae sensu Benson et al. [14]). The reduced tree in Allosauroidea reduced Metriacanthosaurinae (sensu [12]) to *Y*. *hepigensis* and *S*. *dongi* and Carcharodontosaurinae to *Tyrannotitan* and *Mapusaurus*. However, the pruning analysis indicated that only pruning *Streptospondylus* improves Megalosauria node but pruning the rest of “wildcard” taxa does not improve the results. The position of the *Siamraptor* was not affected by both the reduced and pruned trees.

*Siamraptor* presents four allosauroid synapomorphies in this phylogenetic analysis: (1) a projected medial edge of the mandibular glenoid [ch.133 (1)], (2) a pneumatic foramen on the posterior rim of the antorbital fossa of jugal [ch.52 (1)] (see discussion below), (3) an expanded prezygocentrodiapophyseal fossa in dorsal vertebrae [ch.182 (1)]; and (4) a strong constriction in the posterior dorsal vertebrae centrum (hourglass-shaped) [ch.194 (1)]. *Siamraptor* has the following synapomorphy of Allosauria: the presence of fused paradental plates in the maxilla, without replacement grooves and forming a continuous medial lamina [ch.138 (1)]. *Siamraptor* shares with the members of Carcharodontosauria: (1) the presence of two pneumatic foramina oriented anteroventral-posterodorsally in cervical vertebrae [ch.169 (1)], (2) a parallel and sheet-like hyposphene lamina (although this feature is also observed in other allosauroids, as previously mentioned in the description) [ch.187 (1)]; and (3) a reduced and oblique ridge of suprastragalar buttress for the astragalus in the anterior surface of the tibia (shared with *Neovenator* and Carcharodontosauridae, but transformed in a bluntly rounded vertical and medial ridge in megaraptorans [12]) [ch.322 (3)]. Finally, some diagnostic features are obtained based on this phylogenetic analysis: (1) the presence of more than two posterior surangular foramina (an autapomorphy among theropods) [Modified ch. 132 (2)], (2) the presence of marginal enamel wrinkles (shared with Carcharodontosauridae in this phylogeny, although this character actually has a high level of homoplasy because it turns up several times in the Averostra lineage [42]) [ch.143 (2)]; and (3) the flat anterior surfaces of the presacral vertebrae, in contrast to the convex condition in Tetanurae (an autapomorphy among tetanurans) [ch. 156 (0)]. Some symplesiomorphies are determined based on the results of this phylogenetic analysis, such as the posterodorsally-oriented attachment surface of the retroarticular process, which is posteriorly-oriented in Allosauria (except in *Acrocanthosaurus* sensu [41]) [ch.137 (0)]; and the low and blunt epipophyses, as in *Allosaurus* but different in other allosauroids [ch.177 (0)].

**Fig 13:**
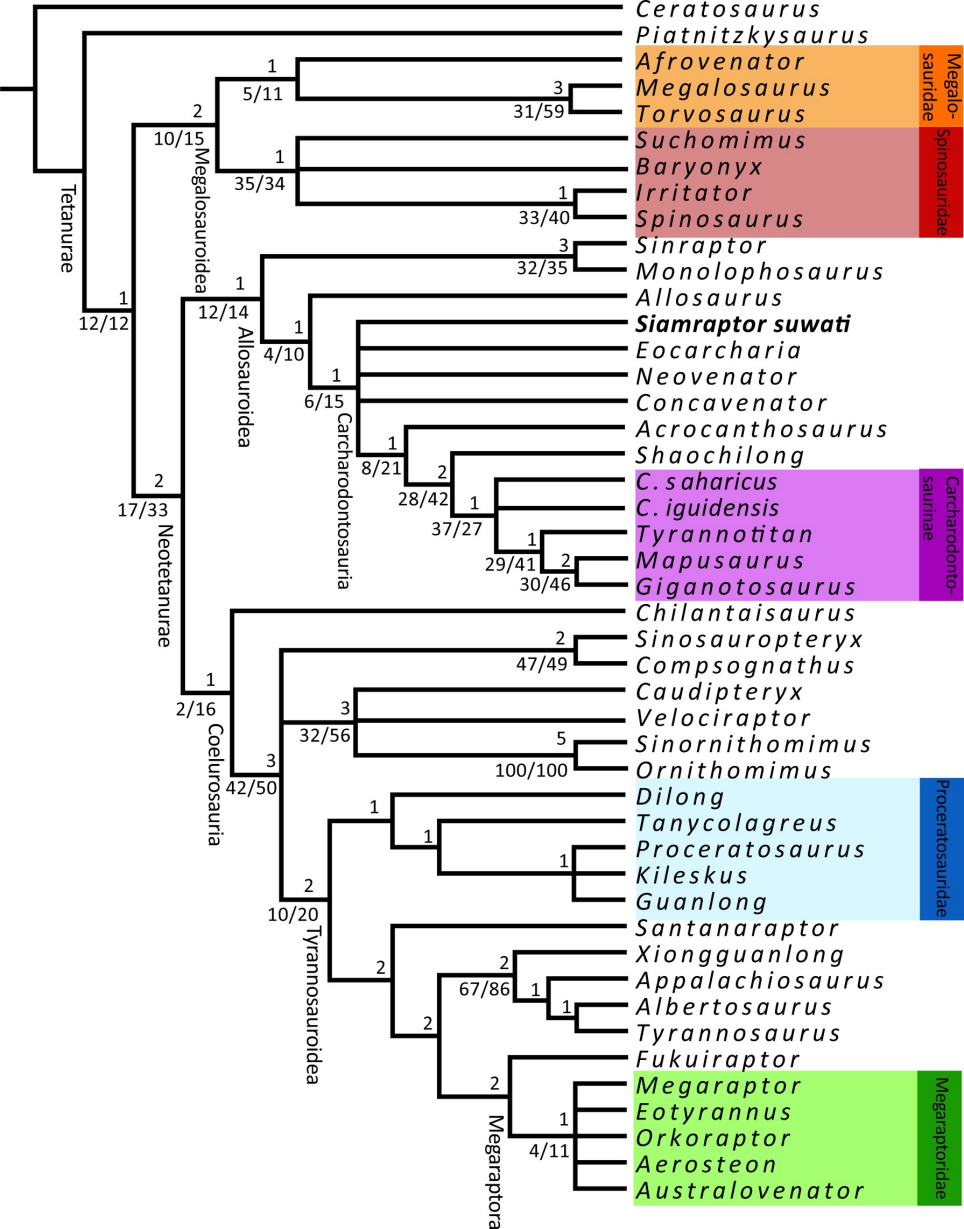
Strict consensus of the phylogenetic analysis based on Porfiri et al. **[13] matrix.** The numbers at the top of the nodes indicate Bremmer support, those at the bottom are bootstraps and jackknife branch support, respectively.

In the data matrix of [13], the results of the heuristic search showed 138 MPTs of 932 steps of length, with CI of 0.371 and RI of 0.664 (Fig 13). *Siamraptor* occupies a similar position within Allosauroidea as in the results obtained in the analysis using the data matrix of [12]. In the strict consensus, the position of *Siamraptor* is more derived than *Allosaurus*, rather nested in a basal polytomy within Carcharodontosauria with *Eocarcharia*, *Concavenator*, and *Neovenator*. This result is obtained mainly by the position of megaraptorids nested in Tyrannosauroidea, unlike the one nested in Allosauroidea in the analysis that follows [12]. The polytomy in the base of Carcharodontosauria is solved in the reduced tree, pruning *Eocarcharia*, *Concavenator*, and *Neovenator*. These specimens, not observed in this agreement subtree, were added one by one to check their phylogenetic position related to *Siamraptor*. However, each taxon included was positioned in the same polytomy. In the reduced tree, *Carcharodontosaurus saharicus* is also pruned from Carcharodontosauridae, and Megaraptoridae is composed by *Megaraptor* and *Eotyrannus*. The pruned analysis indicated that any pruning taxa does not improve the topology of the trees.

Only one allosauroid synapomorphy is present in *Siamraptor* according to this analysis: the presence of the prezygoepipophyseal lamina, a ridge splitting the lateral surface of the neural arch of the cervical vertebra into two [ch.103 (1)]. This feature is also observed in megaraptorids but [13] coded a new character state as a deeper condition for this group. *Siamraptor* shares two features with other members of Allosauria: (1) enamel wrinkles near the denticles [ch.1 (1)]; and (2) a strong medial expansion of the distal medial malleolus [ch.190 (1)]. Based on this analysis, the diagnostic features of *Siamraptor* proposed are: (1) a sub-rectangular alveolar contour of the maxilla, also shared with *Mapusaurus*, *Megalosaurus*, *Tanycolagreus*, and *Eotyrannus* [ch.9 (1)]; (2) a slightly opisthocoelous condition in cervical vertebrae (an autapomorphy of the node of Megalosauroidea+Neotetanurae sensu [13], shared with *Ceratosaurus*, *Piatnitzkysaurus*, and *Dilong* [ch.97 (0)]); (3) the presence of a hyposphene-hypantrum accessory articulation in cervical vertebrae (an autapomorphy among allosauroids, shared with some carcharodontosaurids) [ch.102 (1)], and (4) a subvertical posterior dorsal neural spine (an autapomorphy among allosauroids) [ch. 108 (0)].

Although both analyses differ in the position of Megaraptora (Megaraptoridae sensu [13]), the position of *Siamraptor* as a member of Carcharodontosauria is unambiguously supported by both results.

### Remarks on the allosauroid anatomy

#### Skull anatomy

The tetanuran condition of *Siamraptor* is unambiguous, based on two skull synapomorphies of this group. The presence of a jugal pneumatic foramen has been considered as a tetanuran synapomorphy [56,60]. However, some coelurosaurians appear to lack this structure [82], and it is identified as an allosauroid synapomorphy [12]. Although the morphology and development of this foramen vary within the tetanuran clade, its position in the antorbital fossa of the jugal in *Siamraptor* allows homologizing it to the one described by Sereno et al. [56,60]. Moreover, the presence of a well-developed antorbital fossa in the jugal is a feature shared with some tetanurans [12,67]. In lateral view of the jugal, the ventral margin of the postorbital contact ends above the ventral margin of the orbit in *Siamraptor*. The postorbital contact ends substantially above the ventral margin of the orbit in Avetheropoda [12,14,47]. Besides this lateral contact, the ventral portion of the postorbital also wraps around onto the medial surface and is inserted on a marked notch of the jugal in *Siamraptor*. This notch on the medial surface is also observed in *Sinraptor* [44] and some specimens of *Allosaurus* (e.g. UMNH-VP 9085), in which the ventral end of this medial contact notch is also substantially above the ventral margin of the orbit in *Sinraptor* and *Allosaurus*. In *Siamraptor*, although the lateral surface indicates a dorsally situated end of the postorbital ventral tip, this medial notch runs through the postorbital process down below the orbit, similar to non-avetheropodan theropods such as *Torvosaurus* (“*Edmarka rex*” by [83]; juvenile of *Torvosaurus* by [12]).

The descriptions of several complete allosauroid skulls in recent years (e.g. [41,42,44]) have enabled us to improve the knowledge of the cranial osteology of this widely known theropod group. The presence of two posterior surangular foramina is observed in *Allosaurus* [47] and *Sinraptor* [44], and it has been proposed as a synapomorphy of Allosauroidea that was reverted to the primitive condition in Carcharodontosauridae [47]. However, the basal carcharodontosaurid *Concavenator* also presents two foramina [42], and only *Acrocanthosaurus* lacks the second posterior surangular foramen within Allosauroidea. *Siamraptor* shows an unusual feature relative to the number of posterior foramina in the surangular, due to the presence of four of them. Within Allosauroidea, the skull of *Siamraptor* shows more similarities with members of Allosauria than with basal allosauroids such as metriacanthosaurids. For instance, *Siamraptor* has a well-developed maxillary process in the premaxilla that does not project beyond the posterior margin of the bone, whereas it does in *Sinraptor* [44].

Although the premaxilla with four teeth is the primitive condition for Theropoda [67], this number is variable in several groups of theropods. For example, *Allosaurus* and *Neovenator* have five premaxillary teeth [43,65]. Within Allosauroidea, derived carcharodontosaurids show a plesiomorphic condition that is the presence of only four teeth [11,41]. This condition is unknown in basal carcharodontosaurids due to the absent or incomplete premaxilla in *Eocarcharia* [51] and *Concavenator* [42]. The phylogenetic position in both analyses of *Siamraptor* indicates that the presence of five premaxillary teeth is a parallelism between *Allosaurus* and *Neovenator*, and that the rest of Allosauroidea present the primitive condition. Regarding the premaxilla and maxilla, the interdental plates are fused to form a continuous medial lamina without replacement grooves in *Siamraptor*. This feature has been previously proposed as a synapomorphy of Allosauridae and Carcharodontosauridae [48]. However, other allosaurians also have a continuous medial lamina in the paradental region, such as *Neovenator* [45] and *Fukuiraptor* [23]. Therefore, [12] proposed this feature as a synapomorphy of Allosauria. On the other hand, [13] coded separated interdental plates in *Allosaurus*, although several specimens of *Allosaurus* present this continuous lamina (UMNH-VP 5316, 9168, 9229).

Several carcharodontosaurian features are absent in the skull of *Siamraptor* despite its position as a basal taxon of this more exclusive group. For instance, the ventral portion of the anterior margin of its premaxilla is almost vertically oriented as in basal allosauroids like *Allosaurus* (e.g. BYU 1068; UMNH-VP 6500, 6502, 9248, 9250) and *Sinraptor* [44]. This condition is distinct from the slightly posterodorsally oriented anterior margin in carcharodontosaurians such as *Neovenator* (MIWG 6348) and *Acrocanthosaurus* (NCSM 14345), and thus is proposed as a synapomorphy of Carcharodontosauria within Allosauroidea [11]. This feature is also observed in other theropods such as *Torvosaurus* (BYU 4882; [84]). Within Carcharodontosauridae, the interdental plates in the maxilla are more than twice deeper than anteroposterior wide [11]. Although the interdental plates of *Siamraptor* are deep with respect to their anteroposterior width, their proportions are between 1.19 and 0.96, so they are distinct from the carcharodontosaurid condition. Of course, it is necessary to take account of the absence of the most anterior regions of both maxillae, because the depth of those unknown paradental plates could be even bigger. In *Siamraptor*, the lateral surface of the maxilla is smooth and lacks a strong external sculpturing by groove foramina like in other allosauroids and basal carcharodontosaurids [41,42], unlike in Carcharodontosaurinae [11]. Another absence of derived carcharodontosaurid feature is observed in the orbital margin of the jugal, which is angled posterodorsally in *Siamraptor* whereas is mostly vertically oriented in Carcharodontosaurinae [12]. Meanwhile, the jugal presents a horizontal ridge along its ventral margin in order to contact with the maxilla in *Siamraptor*, and an additional prominent horizontal ridge running from the notch of the quadratojugal processes towards anteriorly throughout the area below the postorbital process. A ridge similar to the latter has also been reported and interpreted as an insertion area for the M. pterygoideus ventralis in carcharodontosaurids such as *Mapusaurus* and *Tyrannotitan* [55], but it is anterodorsally-oriented in these derived carcharodontosaurids. In addition, the jugal of *Siamraptor* presents two ventral foramina posterior to the maxillary contact. Nutrient foramina in the lateral surface of the jugal have been noticed in *Allosaurus fragilis* [43,85] and are observed in the skull of *Allosaurus fragilis* (DINO 2560) in the same position as in *Siamraptor*.

#### Axial anatomy

The prezygapophyses of cervical vertebrae in *Siamraptor* are displaced laterally away from centrum, and the distance between them in each vertebra is wider than the width of the neural canal, a condition that is shared with both abelisaurids [12,69] and tetanurans [12].

Among tetanurans, *Siamraptor* shows several features shared with Allosauroidea and other more exclusive clades. The hyposphene extends ventrally as a sheet with parallel lateral edges in *Siamraptor*. This parallel condition has been defined as a synapomorphy of Carcharodontosauridae [47] and Abelisauridae [12], different from the ventrolaterally divergent triangular shape in many other theropods. However, as abovementioned, other allosauroids also have this parallel and vertical hyposphene laminae in dorsal vertebrae, thus, this condition should be considered as a synapomorphy of Allosauroidea, although it is also observed in some tyrannosauroids [13]. As in Allosauria, *Siamraptor* has the primitive tetanuran condition in the mid-caudal vertebrae, a short rod-like and posteriorly inclined neural spine [67]. This condition contrasts with the morphology observed in metriachantosaurids, which have broadly rectangular and sheet-like neural spine [12].

*Siamraptor* occupies a basal position in Carcharodontosauria as a sister taxon of Neovenatoridae and Carcharodontosaridae. The relationship with these more exclusive clades is based on the sharing of several features with other carcharodontosaurian taxa, such as the presence of two pneumatic foramina in cervical vertebra and a well-developed and subvertically oriented postzygodiapophyseal lamina. The presence of two pneumatic foramina in the cervical centrum is a derived condition in Theropoda [86]. Furthermore, the presence of two anteroposteriorly elongated pneumatic foramina located behind the parapophyses and separated by a thin lamina is a feature observed in Carcharodontosauria, as in neovenatorids such as *Neovenator* [45] and *Aerosteon* [21] and derived carcharodontosaurids like *Giganotosaurus* or *Tyrannotitan* [55]. The pneumatic foramina of *Siamraptor* are not split by a thin oblique sheet of bone as in *Tyrannotitan* and other carcharodontosaurids [55], but both pneumatic foramina are separated by a septum that is more similar to that observed in *Aerosteon* [21]. Regarding the postzygodiapophyseal lamina, it is well developed and subvertically oriented, the diapophysis being extensive and subtriangular in lateral view in the *Siamraptor*, like in other carcharodontosaurians, except *Neovenator* and *Acrocanthosaurus* [13].

Despite these shared features with other carcharodontosaurians, *Siamraptor* lacks pneumatic foramina in the posterior dorsal vertebrae as in most non-carcharodontosaurians. The pneumatic foramina are present in all dorsal vertebrae and some caudal vertebrae in Carcharodontosauria except *Concavenator* [64]. Moreover, the camerate condition is visible in *Siamraptor* due to the presence of cavities consisting of several large chambers that are not further subdivided [62]. This type of internal pneumatic cavities is present in most tetanurans but is distinct from those camellate-type vertebrae of derived carcharodontosaurids and other carcharodontosaurians [45,58]. Moreover, derived carcharodontosaurids like *Tyrannotitan* (MPEF-PV 1157) and *Giganotosaurus* (MUCP-Ch 1) have a strong opisthocoelic condition in the cervical vertebrae. However, the cervical vertebrae of *Siamraptor* have an almost flat anterior articular surface, even more than those observed in *Allosaurus* (e.g. UMNH-VP 8348, 8352, 8487); and rather similar to the cervical vertebrae of *Sinraptor* [44].

The basal position within Allosauria is also supported by the offset in the positions of the articular surfaces of the cervical vertebrae. In *Siamraptor*, the posterior articular surface is strongly ventrally projected with respect to the anterior one. Although this condition of the cervical vertebrae is commonly seen in Dinosauria [87], among allosauroids this condition is only observed in basal allosaurians such as *Allosaurus* (UMNH-VP 8352, 8487, 8519, 10192) and some neovenatorids [45,88]. The offset is substantially reduced in the cervical centra of carcharodontosaurids [11,56].

Some symplesiomorphic conditions shared with other allosauroids are observed in the axial skeleton of *Siamraptor*, including the cervical epipophyses and the dorsal neural spines. A long cervical epipophysis was proposed as a synapomorphy of Carcharodontosaridae [55]; however, this feature is also well developed in other carcharodontosaurians like *Neovenator* [45], other allosauroids like metriacanthosaurids [44], and other theropods like ceratosaurians [69], indicating a long epipophysis as a derived condition in theropods. Therefore, *Siamraptor* shows a primitive condition due to the short epipophyses in its cervical vertebrae, a symplesiomorphy also observed in *Allosaurus*. Although not complete, *Siamraptor* also shows a primitive condition in the dorsal neural spine of the dorsal vertebra due to its vertical orientation, unlike the anteriorly-inclined neural spine in some allosauroids [58].

#### Appendicular anatomy

Two tetanuran conditions of the ischium are observed in *Siamraptor*. One of them is a reduction of the antitrochanter in the ischium. Conversely, the primitive condition in theropods is the presence of a large and well-developed antitrochanter in the ischium [61,89,90]. In addition, although the ischial obturator process is not complete, a ventral notch seems to be developed in *Siamraptor*. The presence of this notch is another tetanuran synapomorphy [60].

The suprastragalar buttress for the astragalus is oblique in *Siamraptor* like in other tetanurans, but it is more reduced and rounded, like in some carcharodontosaurians such as *Neovenator* and derived carcharodontosaurids [12]. However, this morphology is distinct from megaraptorans in that it is transformed into a vertical ridge on the medial side [12]. Another feature in the tibia of *Siamraptor* that is shared with other carcharodontosaurians is the strong distal extension of the lateral malleolus beyond the medial malleolus. This condition differs from those observed in *Allosaurus* and primitive allosauroids, in which the distal extension of the lateral malleolus is absent or indistinct [11]. Within Carcharodontosauria, *Siamraptor* has a medially oriented medial malleolus of the tibia as in Carcharodontosauridae [47].

Regardless of the controversial phylogenetic position of Megaraptora in Allosauroidea [14] or Tyrannosauroidea [13], *Siamraptor* presents a feature in the manual ungual that is similar to megaraptorans. This feature is based on a new character [14] about the proximal height and width ratio of the manual unguals. The transversally narrow condition, observed in some megaraptorans and coelurosaurs, was defined with a ratio of 2.4, in contrast to the 2.0 ratio of the other theropods as the basal condition. Despite a slight incompleteness of the proximal end, the ratio is 2.26 in the manual ungual of *Siamraptor*, showing at least an intermediate state between the megaraptoran condition and the primitive condition for theropods.

Although *Siamraptor* shares several appendicular features with the lineage of carcharodontosaurians, this new taxon also shows differences against the members of more exclusive clades. For instance, a peg-and-socket-like iliac articulation in the ischium is a synapomorphy of Carcharodontosauridae [12], and it is also observed in the neovenatorid *Siats meekerorum* [68] and some abelisauroids [69]. *Siamraptor* differs from this derived condition because it presents a flat surface, like most theropods.

Some primitive conditions are observed in the ischium of *Siamraptor*, such as the absence of a posteriorly-oriented flange in the iliac peduncle, which is a feature observed in *Allosaurus* and Carcharodontosauridae [11]. In addition, the distal end of the ischium is rounded or slightly expanded. This feature is considered as a primitive condition in Theropoda [67] that is also retained in tetanurans and tapered distally into a point in coelurosaurs [67,69]. The rounded distal end of the ischium of *Siamraptor* is distinct from the well-developed and expanded ischial boot observed in some carcharodontosaurians such as *Neovenator* [45] and *Concavenator* [72] as well as other allosauroids like *Yangchuanosaurus* [91]. Despite these primitive features in the ischium, a metriacanthosaurid condition is present in the tibia. The notch observed in the medial part of the posterodistal margin in *Siamraptor* seems to be homologous to the deep pit for the articulation with the astragalus described in *Sinraptor* [44], and observed in a metriacanthosaurid theropod from Thailand [73].

#### Comparison with Asian allosauroids

Allosauroidea is widely represented in Asia by several taxa, especially by members of Metriacanthosauridae and Carcharodontosauria. These clades were the apex predators from Late Jurassic to mid-Cretaceous [5]. *Siamraptor* is proposed here as a new Asian allosauroid from the Early Cretaceous of Thailand, which belongs to the less inclusive clade Allosauria (Allosauridae + Carcharodontosauria sensu [12]).

The Early Cretaceous of Thailand has also yielded other allosauroids, *Siamotyrannus isanensis* [7] and an isolated small fragment of an indeterminate carcharodontosaurid. Originally, *Siamotyrannus* was defined as a primitive member of Tyrannosauroidea [7]. However, subsequent studies have reinterpreted this taxon as a member of Allosauroidea (e.g. [1,11]) and the phylogenetic analysis of Carrano et al. [12] proposed it as a member of Metriacanthosauridae. *Siamotyrannus* (PW9-1) is represented by a pelvic girdle, the last dorsal vertebra, a sacrum and anterior caudal vertebrae in articulation. Therefore, *Siamraptor* shares only the ischium and the posterior dorsal vertebra with *Siamotyrannus*. Even though several parts are missing, such as the obturator process in both taxa, the mid-shaft in *Siamraptor* and the distal end in *Siamotyrannus*, the ischium can be compared in several points. For instance, the ischium of *Siamraptor* shares a symplesiomorphy with the most primitive allosauroids including *Siamotyrannus*, namely, the absence of a posteriorly oriented flange in the iliac peduncle. On the other hand, the pubic peduncle of *Siamotyrannus* is anteriorly elongated, which makes the acetabulum concavity wider than in *Siamraptor*. Moreover, the proximal end of *Siamotyrannus* is dorsoventrally shorter than the one in *Siamraptor*. The proximal obturator notch is dorsoventrally narrow in *Siamotyrannus*, distinct from that of *Siamraptor*, in which the notch is widely opened dorsoventrally. A diagnostic feature is a curved shaft of the ischium [7], which seems to be straight in *Siamraptor*. The distinct crest along the posterior margin of the ischial shaft is present in *Siamotyrannus* (PW9-1), as in other metriacanthosaurids [44,91–93] except *Metriacanthosaurus* (Fig 16 in [94]). In *Siamraptor*, only the proximal end of this crest is observed in the proximal shaft, and it is clearly absent in the distal part, so it seems to be smaller than those of metriacanthosaurids. In *Siamotyrannus*, only the centrum of the posterior dorsal vertebra is preserved and exhibits the hourglass-shape, as seen in *Siamraptor* and other allosauroids. The pneumatic foramen is absent in both taxa, as in other basal allosauroids. Regarding the other allosauroid of Thailand, a fragmentary and non-diagnostic maxilla is reported as an indeterminate carcharodontosaurid from the Barremian Sao Khua Formation [25]. This maxilla represents a posterior part of the right maxilla; for that reason, it is possible to compare it with *Siamraptor*. Both specimens have several common features with carcharodontosaurians in their maxillae: fused interdental plates forming a single lamina with faint suture lines; tall interdental plates that increase in height towards the anterior end; the presence of the groove for the dental lamina; and a swollen medial wall dorsal to the groove for the dental lamina. There are only two differences between both specimens: in *Siamraptor*, the maxillary alveoli are more rectangular and lateromedially compressed in ventral view, and the superior labial foramina are much smaller and they are more open laterally than ventrally. The fragmentary nature of Sao Khua carcharodontosaurid makes further comparison difficult, but it is not ruled out that both specimens could be related to each other in spite of the temporary discordance.

Other Asian metriacanthosaurids are *Yangchuanosaurus shangyouensis* [95] (including *Y*. *magnus*, sensu Carrano et al. [12]), ‘*Y*.’ *hepingensis* Gao [96], ‘*Szechuanosaurus*’ *zigongensis* Gao [91] (*Y*. *zigongensis* sensu Carrano et al. [12]), and *Sinraptor dongi*, all from the Late Jurassic of China. The distal end of the ischium is separated in *Siamraptor* as in most tetanurans; however, it is fused in Metriacanthosauridae [12,92]. The epipophyses of the cervical vertebrae are extremely elongated and robust in metriacanthosaurids such as *Sinraptor* [44], *Y*. *shangyouensis* [92] and *Shidaisaurus* [93], distinct from the blunt and reduced epipophyses of the cervical vertebra seen in *Siamraptor* and *Allosaurus* (e.g. UMNH-VP 8348, 8352, 10192; BYU 12023). A single pneumatic foramen excavates the cervical centrum of *Sinraptor* [44], ‘*Y*.’ *hepingensis* [96], ‘*S*.’ *zigongensis* [91], in contrast to the two anteroposteriorly elongated pneumatic foramina developed in the cervical vertebrae of *Siamraptor*. In dorsal vertebrae, the hyposphene lamina diverges ventrolaterally in *Sinraptor* [44] and other metriacanthosaurids [12], distinct from the vertical and parallel hyposphene of *Siamraptor*. Despite the abovementioned differences against metriacanthosaurids, *Siamraptor* also shares some similar features with some of them. For instance, a posterodorsally oriented retroarticular process in the articular is seen in *Sinraptor* and *Y*. *shangyouensis* [12], although *Acrocanthosaurus* also presents this character [41]. Moreover, a vertical dorsal neural spine is shared with *Y*. *shangyouensis* [92] and, as discussed above, a deep medial pit in the posterodistal end of the tibiae is shared by *Siamraptor*, *Sinraptor*, and a metriacanthosaurid from Thailand [73].

*Kelmayisaurus petrolicus* [97] is another allosauroid from the Early Cretaceous of China that has been identified as a carcharodontosaurid [24]. There is only one autapomorphy for *Kelmayisaurus* in the dentary, so that the absence of the dentary in *Siamraptor* makes it difficult to carry out comparisons that allow determining a synonymy between both taxa. Contrarily, both specimens have fragments of the maxilla. Even though the parts preserved in those fragments do not correspond with each other, it is possible to compare several characteristics. The alveoli of *Kelmayisaurus* are oval [24] in contrast to the rectangular ones in *Siamraptor*. Furthermore, *Kelmayisaurus* (IVPP 4022) lacks the strongly marked medial wall over the interdental plates seen in *Siamraptor*, although the maxilla of the former is heavily abraded [24].

The mid-Cretaceous is represented by the carcharodontosaurid *Shaochilong maortuensis* [98] and the neovenatorid *Chilantaisaurus tashuikouensis* [99]. Regarding the first taxon, only the maxilla and the caudal vertebra are comparable with *Siamraptor*. The maxilla of *Shaochilong* is complete and it is characterized by two autapomorphies: the paradental groove is absent; therefore, the interdental plates and the medial wall are not clearly separated; and the interdental plates are excavated by several dorsoventrally-oriented grooves [5]. *Siamraptor* lacks those dorsoventrally-oriented grooves, although they are more anteriorly located in *Shaochilong*. The most striking difference is the absence of a clearly distinct medial wall in the maxilla, because *Siamraptor* has a well-developed and strongly marked ridge in both the dorsal and ventral margins of the medial wall. The teeth are also different, *Siamraptor* presents teeth mesiodistally thicker and more labiolingually compressed. Moreover, *Shaochilong* seems to lack marginal undulations, distinct from those observed in *Siamraptor*. The middle caudal vertebra is known for both taxa. *Siamraptor* shows anteriorly projected prezygapophyses, unlike the short ones that barely reach the anterior border of the centrum in *Shaochilong*. Conversely, the postzygapophyses are strongly projected in *Shaochilong*, further posterior to the centrum, unlike the shortly projected ones in *Siamraptor*.

*Chilantaisaurus* and *Siamraptor* can be compared only on the distal end of the tibia. The main difference is the astragalar buttress, which is a robust longitudinal ridge along the medial margin of the tibia in *Chilantaisaurus* [6], and a reduced proximolaterally-oriented ridge in *Siamraptor*.

*Fukuiraptor kitadaniensis* [23] is the only allosauroid known from the Early Cretaceous Japan. It can be compared with *Siamraptor* on the maxilla, cervical and dorsal centrum, and the ischium. In dorsal view, the bony plate comprising the antorbital fossa is thick in *Fukuiraptor* (FPDM-V43) in contrast to that of *Siamraptor*. In lateral view, the superior labial foramina are present as a row just below the ventral rim of the antorbital fossa, whereas they are located along the ventral margin of the lateral surface in *Siamraptor*. Moreover, the area ventral to the antorbital fossa is dorsoventrally narrower than in *Siamraptor*. The medial wall is dorsoventrally narrower than that of *Siamraptor*. The pneumatic foramina of the cervical vertebra are developed but not extremely, unlike *Siamraptor*. The centrum is anteroposteriorly elongate, in contrast to the short one of *Siamraptor*. The ventral surface is concave between the parapophyses whereas it is almost flat in *Siamraptor*. a step-like lateroventral margin of the postzygocentrodiapophyseal fossa is absent, unlike *Siamraptor*. On the dorsal centrum, although that of *Fukuiraptor* also seems to belong to the posterior dorsal vertebra in comparison with *Allosaurus*, the ventral surface is smoothly rounded and lacks the midline ridge, which is present in *Siamraptor*.

### Skeletal pneumaticity compared with *Aerosteon* and *Murusraptor*

Pneumaticity in bones, especially in axial elements, has evolved independently in sauropodomorphs and basal theropods [21]. This pneumaticity is represented by several distinct structures like pneumatic foramina and pneumatopores in several saurischian taxa in the fossil record (e.g. [21,22,100,101]).

*Siamraptor* is characterized by cranial and axial bones that are remarkably pneumatic. This pneumaticity is comparable with those observed in other two allosauroids, *Aerosteon* and *Murusraptor*, although both taxa could have another phylogenetical interpretation as tyrannosauroids (see “Phylogenetic analysis”).

The surangulars of *Siamraptor* show multiple striking foramina, some of them could be related with an unusual surangular pneumaticity. The most striking feature is the presence of large, smooth-walled external openings separated by septa in the posterior part of the surangular, which are observable in anterior view, and the presence of an oval concavity posterior to the lateral shelf. The pneumaticity in the mandible is also reported in *Murusraptor* and derived tyrannosauroids. The articular of *Murusraptor* is strongly pneumatized, but the condition is unknown in the surangular [22]. Gold et al [102] argued that the surangular pneumaticity is unusual in theropods, but it is described in derived tyrannosauroids which share several characteristics with that observed in *Siamraptor* as follows: 1) the pneumaticity in *Siamraptor* and derived tyrannosauroids, such as *Tyrannosaurus*, is extremely related to the posterior area of the surangular, close to the articular. 2) although, as mentioned by these authors, the surangular foramina are not necessarily referred to a pneumatic opening, the authors also suggested that the enlargement of the surangular foramen of derived tyrannosauroids could be associated to the surangular pneumaticity. In *Siamraptor*, there is not a strongly enlarged surangular foramen, but there is a striking increase in the number of these foramina and, even, the posteriormost one is slightly wide compared to those in other allosauroids.

The axial pneumaticity is the most developed of the *Siamraptor* features. The centrum of the cervical vertebrae is strongly excavated by pneumatic foramina that penetrate through the lateral surfaces broadly, separated by septa as those observed in the mid cervical centra of *Aerosteon* [21]. There are also camerate structures within the centrum, with huge camerae separated by septa; and other smaller and oval camerae penetrating the neural arch. Wedel [103] proposed that the large camerae in a camerate structure may bifurcate to produce successive generations of smaller camerae. The centrum of some vertebrae of *Murusraptor* has also this camerate structure [22], distinct from the camellate structure proposed in *Aerosteon* [21] and in other carcharodontosauria (e.g. Brusatte et al., 2008). In the anterior cervical vertebra of *Siamraptor*, there is a small foramen within the prezygocentrodiapophyseal fossa. This type of pneumatopores invading the neural arch through the vertebral fossa is also observed in cervical and dorsal vertebrae of *Aerosteon* (MCNA-PV-3137) and *Murusraptor* (MCF-PVPH-411, [22]). Moreover, the centrodiapophyseal and postzygocentrodiapophyseal fossae in the cervical and dorsal vertebrae of *Siamraptor* are extremely excavated and they connect with the huge internal camerae of the neural arch. Based on the Pneumaticity Profile of the neural arch [104], the presence of these foramina or fossae that connect with the internal camerae are an unambiguous sign of pneumaticity. In the neural spine of cervical and dorsal vertebrae of *Siamraptor*, it is also observed that a pair of pneumatopores pierce the base of the spine bilaterally. Similar pneumatopores are also present in the atlas of *Aerosteon* [21] and in the caudal vertebra of *Murusraptor* (MCF-PVPH-411), and its presence also indicates the formation of an air sac in the base of the neural spine. Finally, the neural spine filled by a huge lumen is slightly similar to that described in *Aerosteon* [21], although it is shorter than the latter due to the development of a strong metaplastic scar. The axial pneumaticity is attributed to cervical, thoracic or abdominal air sacs in non-avian saurischian dinosaurs like in living birds [21,105,106]. The pneumaticity observed in the surangular, articular, and axial skeleton of *Siamraptor* is only superficially described so far; for that reason, further studies about the pneumatization of this theropod, using a CT scan, should be performed in order to evaluate the pneumatized structures.

### Paleobiogeographic implications

The major clades of Allosauroidea seem to have widely global distributions with few time intervals where samples of allosauroids are known [14]. Metriacanthosauridae, the most basal clade within Allosauroidea [12], is represented mostly by Asian taxa, except *Metriacanthosaurus* from the Late Jurassic of England [107]. Allosauridae, the sister taxon of Carcharodontosauria [12], is composed of *Allosaurus + Saurophaganax*, both from the Upper Jurassic of USA, and the genus *Allosaurus* also have representatives from the Lusitanian Basin in Portugal. Most genera known from the Lusitanian Basin record have closely related taxa in the Morrison Formation record, although some taxa closely related to those of North America have been reinterpreted as independent Portuguese species, as is the case of *Allosaurus europaeus* [108]. This reinterpretation implies an emerging vicariant evolution of the Late Jurassic theropod faunas [26]. Carcharodontosauria is composed of two more exclusive clades, Neovenatoridae and Carcharodontosauridae, and both clades have Asian representatives. Neovenatoridae comprises two Asian taxa, *Fukuiraptor* from the Lower Cretaceous of Japan and *Chilantaisaurus* from the mid-Upper Cretaceous of China. Carcharodontosauridae also comprises two taxa from China, namely *Kelmayisaurus* from the Lower Cretaceous and *Shaochilong* from the mid-Upper Cretaceous. However, representatives of both clades were more widely distributed in Laurasia landmasses during the Early and mid-Cretaceous (e.g. [23,56,65,68,74,75]), and mostly distributed in Gondwana during the Late Cretaceous (e.g. [22,48,54,55,109]). The earliest carcharodontosaurian from Laurasia was recently reported from fragmentary material from the Upper Jurassic of the Lusitanian Basin, Portugal [26]. The presence of these taxa in Gondwana and Laurasia during the Upper Jurassic implies that this group was early spread in both landmasses. Under this scenario, the presence of a new basal carcharodontosaurian from the Lower Cretaceous of Thailand supports an extension of the record in the Laurasian landmasses during the earliest stage of the evolutionary history of this clade. An early widely distribution of the most exclusive clade Carcharodontosauridae has been previously suggested [110]. Posteriorly, a near-cosmopolitan distribution is achieved by major allosauroids clades [14]. This broad geographical distribution occurred in other major theropod clades, early in their geological history [12]. The most recent studies propose a model of distribution of late Mesozoic taxa that supports these previous hypotheses [27, 30]. The network models [28] show a cosmopolitan distribution of theropod taxa through mid-Mesozoic, including Early Cretaceous, where there were huge fauna interchanges between America, Asia, and Europe. The results concur with the possibility of full network connectivity between Laurasian landmasses persisting up until Berriasian–Barremian, results also detected by Ezcurra and Agnolin [20]. This situation matches with the high distribution of Carcharodontosauria during the Late Jurassic and Early Cretaceous, with the presence of basal members of this clade in Europe and Asia.

## Conclusions

A new carcharodontosaurian theropod, *Siamraptor suwati*, is described based on isolated cranial and postcranial remains from the Lower Cretaceous Khok Kruat Formation. *Siamraptor* is diagnosed by eight characters in cranial and axial elements, and it also exhibits several carcharodontosaurian synapomorphies such as two pneumatic foramina oriented anteroventral–posterodorsally in cervical vertebrae, a parallel and sheet-like hyposphene lamina, and a reduced and oblique ridge of suprastragalar buttress for the astragalus in the tibia. *Siamraptor* is also characterized by remarkable pneumaticity in cranial and axial bones, which is comparable with those observed in several other carcharodontosaurians, although those taxa could have another phylogenetical interpretation as tyrannosaurids. Both phylogenetic analyses using two independent datasets locate *Siamraptor* as the most basal member of Carcharodontosauria, which also means that this taxon is the first definitive carcharodontosaurian theropod from Southeast Asia. The presence of *Siamraptor* in this area indicates an extension of the record in the Laurasian landmasses during the earliest stage of the evolutionary history of Carcharodontosauria.

## Supporting information

S1 Appendix Phylogenetic and data matrices information. (DOCX)
