# Supplementary material for: A new carcharodontosaurian theropod (Dinosauria: Saurischia) from the Lower Cretaceous of Thailand
Source: PLoS One. 2019 Oct 9;14(10):e0222489. doi: 10.1371/journal.pone.0222489 (PMC6784982; doi:10.1371/journal.pone.0222489)
Supplement: S1 Appendix — (DOCX) [file pone.0222489.s001.docx]

**S1 Appendix. Phylogenetic and Data matrices information**

**Additional state of character to the Carrano et al. [12]**

132. **Surangular,** number of posterior surangular foramina: one (0), two (1), more than two (2)

State 1 proposed by Carrano et al. [12] describes the condition in some allosauroids of the presence of two posterior surangular foramina. However, *Siamraptor* *suwati* presents four of these foramina, condition that is proposed here as an additional state of character.

**Coded characters in *Siamraptor suwati***

*Carrano et al.* [12] *data matrix*

*Siamraptor suwati* 00???00?00 0???????00 ?????????? ???0?????? ?????????? ?11100??0? ?????????? ?????????? ?????????? ?????????? ?????????? ?????????? ?????????? 12111?0100 0020001100 00?0?01001 ????????11 10000101?0 001???1?00 00?11????? ??????0??? ?????????? ?????????? ?????????? ?????????? ?????????? ?????????? ?????????? ?????????? ???011??00 ?????????? ???????0?? ?3???????? ?????????? ?????????? ?

*Porfiri et al.* [13] *data matrix*

*Siamraptor suwati* 10?1??0010 000?2????? ???01?10?? ?1???????? ?????????? ?????11?10 11???????? ?????????? ?????1???? ??????031? 0111?100?? ?0?0?????? ?????????? ?????????? ???0?????? ?????????? ???1?0??10 ?????????? ??????20?1 1????????? ???????1?? ??0??????? ???0?????? ?????????? ???1?????? ??1???000? ?????????? ?0?200?001 1???

**Agreement subtree in *Carrano et al.* [12] analysis**

**Agreement subtree in *Porfiri et al.* [13] analysis**
